# Root exudates and microbial community structure characteristics of mango under soil borne diseases

**DOI:** 10.3389/fmicb.2025.1627112

**Published:** 2025-07-10

**Authors:** Yongjun Xie, Wenlian Qin, Mengjia Wang, Xiaozhuo Pan, Xiaojie Qin, Yibing Wang

**Affiliations:** Guangxi Key Laboratory for Polysaccharide Materials and Modifications, College of Marine and Biotechnology, Guangxi Minzu University, Nanning, China

**Keywords:** *Mangifera indica* L, soil borne disease, soil microorganism, Illumina MiSeq, root exudate, soil physicochemical properties

## Abstract

**Introduction:**

As the years of mango cultivation progress, pathogens invade the soil, leading to the development of soil borne diseases. These diseases not only change the physical and chemical properties of the soil but also influence the diversity and composition of soil microbes, ultimately impeding the development of the mango industry. In view of this, this study aimed to explore the correlations among the physical and chemical properties of mango root soil, root exudates, soil microbial community and soil borne diseases.

**Methods:**

Healthy, diseased and severely diseased mango root soil samples were taken as the investigation objects. The main research methods were: (1)Testing seven soil physicochemical properties, such as total phosphorus and total potassium, in rhizosphere soil. (2) We determined the phenolic acid content in mango rhizosphere soil using high-performance liquid chromatography (HPLC). (3) Soil microbial communities were analyzed using second-generation high-throughput sequencing technology. (4) The characteristics and response mechanisms of changes in soil microbial community structure were analyzed using multivariate statistical methods, such as redundancy analysis (RDA) and correlation analysis, in combination with physical and chemical environmental factors. (5) PICRUSt2 analysis of microbial community function under soil borne diseases.

**Results:**

Soil borne disease had profound impacts on soil physicochemical properties, root exudates (phenolic acid) and microbial community structure. On one hand, with the development of soil—borne disease, the mango’s ability to absorb foreign nutrients is weakened, leading to the accumulation of nutrients in the root soil, which significantly increases total phosphorus, total potassium, alkaline hydrolysis nitrogen, acid—soluble phosphorus, available potassium, organic matter and pH value. On the other hand, soil borne disease also increased the secretion of phenolic acid in mango root, with significantly increased concentrations of vanillic acid, ferulic acid, salicylic acid and coumaric acid. High-throughput sequencing results showed that soil-borne diseases were followed by a decrease in bacterial diversity, an increase in fungal diversity, and the accumulation of pathogenic microorganisms such as Fusarium in the soil. In addition, the physical and chemical properties of the soil as well as phenolic acid exudates also influenced microbial community functioning, particularly with respect to genetic information processing, metabolism and biological systems.

**Discussion:**

In this study, we investigated the mechanism of soil-borne diseases in mango by studying the response mechanism of soil-borne diseases with root secretion and microbial community. It provides theoretical support for the sustainable development of mango industry.

## 1 Introduction

Mango (*Mangifera indica* L.) is a fruit of substantial economic relevance, extensively cultivated in tropical and subtropical areas and valued globally for its distinctive flavor and high nutritional benefits (Anaya-Esparza and Montalvo-González, 2019). As the scale of mango cultivation expands, soil borne diseases that lead to continuous cropping challenges have become prevalent, significantly hindering the development of mango industry (Tsao et al., 1994; Li and Zhang, 2023). Reports indicate that due to mango’s prolonged cultivation cycle, pathogens such as bacteria, fungi, and nematodes can easily proliferate in the soil and infect the plants via interactions between the soil and the root system (Sulaiman and Bello, 2024). When pathogens proliferate extensively, they result in considerable losses. Notable soil borne diseases affecting mango include root rot, fusarium wilt, stem rot, all of which adversely impact both fruit yield and quality (Widiastuti et al., 2023; Zakaria, 2023). Consequently, investigating the mechanisms of formation and effective prevention and control strategies for soil-borne mango diseases is crucial for fostering the sustainable growth of the mango industry.

Numerous studies have indicated that the degeneration of rhizosphere soil microflora along with the disruption of microbial community structure are significant contributors to the prevalence of soil borne diseases (Lu et al., 2022; Zhang et al., 2023). Reports reveal that following the sustained cultivation of crops such as ginseng and soybean, rhizosphere microorganisms gradually transitioned from a “bacterial” to a “fungal” dominance, resulting in an increase in root pathogenic fungi and the subsequent emergence of soil borne diseases (Chang et al., 2022; Bi et al., 2023). Prolonged monoculture practices also lead to alterations in the rhizosphere microbial community structure, characterized by the depletion of beneficial microorganisms, a reduction in community diversity, and diminished disease resistance, ultimately affecting the quality of the soil microenvironment (Li et al., 2023). Ongoing crop rotations frequently result in a decline in the populations of bacterial genera that are advantageous for plant development, such as Acinetobacter, Arthrobacter, and slow-growing Pseudomonas (He et al., 2023).

As research continues to advance, an increasing number of scholars assert that the disruption of microbial community structure, the build-up of root exudates, and the degradation of soil’s physical and chemical properties are primary contributors to the challenges associated with continuous cropping (Gong et al., 2023; Miao et al., 2023). Root exudates, which comprise a diverse array of amino acids, sugars, organic acids, and growth factors, serve as a conduit for interaction between plants and their surrounding environment (Canarini et al., 2019). Under conditions of abiotic stress, plant roots tend to release secondary metabolites that alter the composition of microbial communities (Sharma et al., 2023). These compounds possess the capability to recruit, repel, stimulate, inhibit, or even eradicate various organisms and pests (Lamichhane et al., 2024). The accumulation of root secretions in the soil can significantly influence its characteristics. Such secretions release autotoxins that impact soil microorganisms, leading to alterations in soil’s physical and chemical properties (Balyan and Pandey, 2024), modifications in microbial community composition, imbalances in nutrient and enzyme activities, reductions in beneficial microbial populations, and an increase in pathogenic potential (Liao and Xia, 2024). In summary, investigating the dynamics of root exudates and their interactions with microbial communities and soil physicochemical properties is crucial for the comprehension and management of soil borne diseases.

The existing literature on the rhizosphere soil of mango trees is sparse, and the relationships between microorganisms, root exudates, and soil nutrients are not well established. To address this gap, we selected three categories of mango root soils influenced by soil-borne pathogens to varying extents: healthy, diseased, and severely diseased. We utilized high-throughput sequencing techniques to efficiently identify soil microorganisms, employed high-performance liquid chromatography to analyze compounds released by mango roots, and performed assessments of the physical and chemical characteristics of the soil. The aims of our investigation were: (1) to elucidate the impact of soil borne diseases on the composition of rhizosphere microbial communities; (2) to examine how soil borne diseases affect the physical and chemical attributes of mango soil and root exudates; and (3) to uncover the complex interactions among root secretions, microorganisms, and soil characteristics. These methodologies will enhance our comprehension of the rhizosphere ecosystem and clarify the mechanisms underlying soil borne diseases in mangoes, ultimately contributing to the sustainable advancement of the global mango industry.

## 2 Materials and methods

### 2.1 Site description and sample collection

The soil samples for this research were gathered on October 6, 2023, from the primary demonstration zone of the mango poverty alleviation initiative situated in the Tianyang District of Baise City (23°80′89.86″N, 106°96′19.31″E). The region is characterized by mountainous terrain with elevations ranging from 250 to 800 meters, predominantly featuring karst formations. It is classified under the southern subtropical monsoon climate, exhibiting an average annual temperature between 18 and 22°C, abundant sunlight, significant warmth, and average precipitation ranging from 1,100 to 1,350 mm annually. The main soil types identified include red soil, lateritic red soil, calcareous soil (rock), and alluvial soil. Soil samples were collected from 12 designated sites. The average total phosphorus (TP) content recorded was 0.7825 g/kg, the mean total potassium (TK) was 18.71 g/kg, the average alkaline hydrolysis nitrogen (AN) was 108 mg/kg, the mean acid-soluble phosphorus (AP) was 91.9425 mg/kg, and the average available potassium (AK) was 165.69 mg/kg. Additionally, the mean organic matter (OM) content was found to be 0.81 mg/kg, with soil pH exhibiting weak acidity at a value of 6.105.

**Figure 1:**
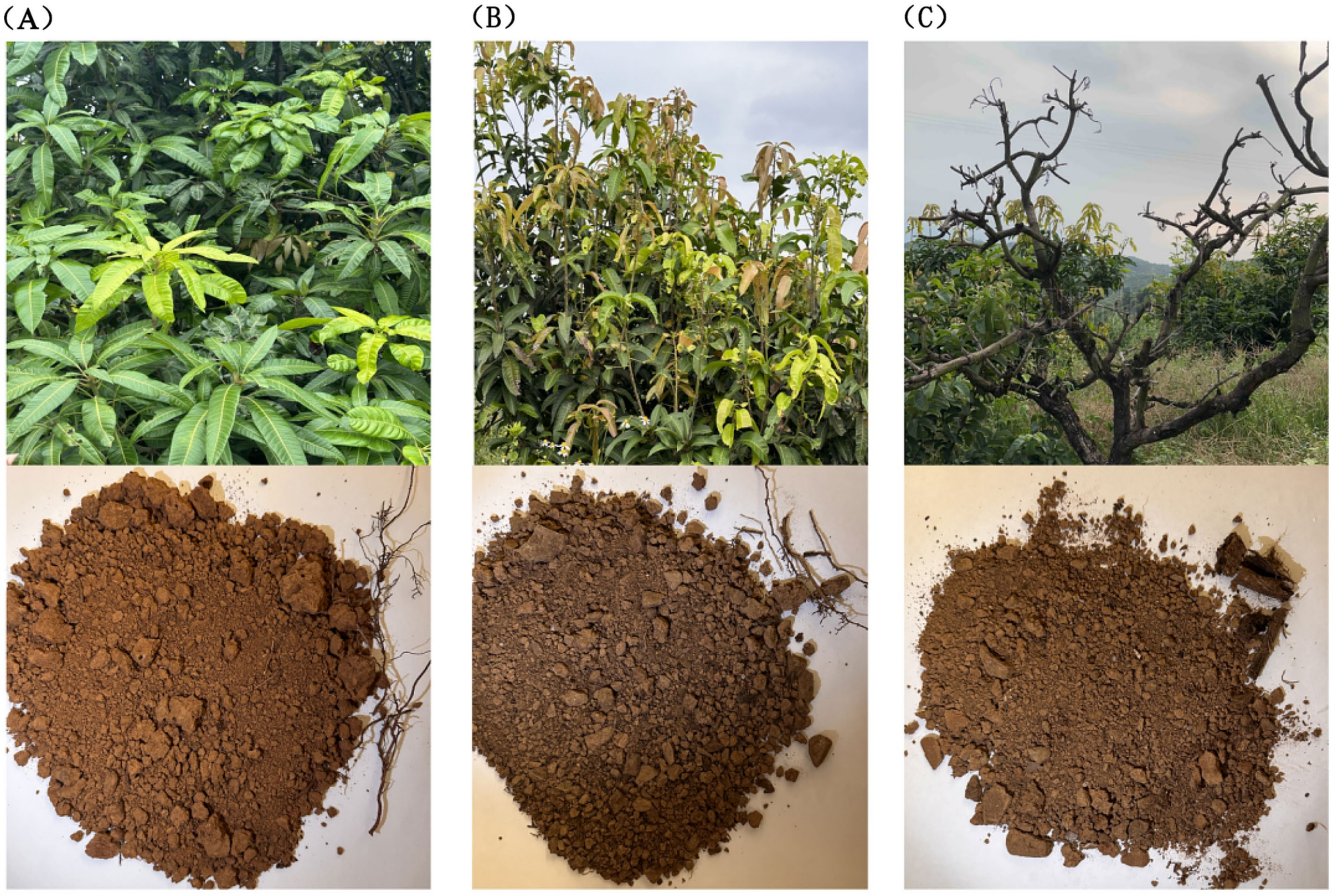
Growth status of mango trees and soil collected from their roots. **(A)** The TN1 sample, the leaves are green, the growth is healthy, the root system is strong, and the soil is yellow. **(B)** The TN2 sample, the leaves of the fruit trees are yellow, the roots are seriously affected by root rot, and the soil is yellow-black. **(C)** The TN3 sample, the branches have completely rotted, the roots are very badly affected by root rot, and the soil is yellow.

This study concentrates on root rot disease, the most common soil-borne affliction experienced by mango trees. We selected three categories of trees for analysis: healthy (TN1), diseased (TN2), and severely diseased (TN3), all maintained under a consistent 10-year forest management regime (Figure 1). The tree species were categorized as follows: TN1 designates healthy mango trees exhibiting robust foliage and well-established root systems; TN2 refers to the diseased cohort, characterized by yellowing branches and leaves, indicating clear evidence of pest infestations and disease, along with slightly blackened and decaying roots; TN3 represents the severely infected group, marked by decaying branches and stems, lack of foliage, and decomposing roots. The five-point sampling method was employed, removing the top 10 cm of soil along with fallen leaves and branches from mango trees afflicted with various diseases. Subsequently, the mango roots were enveloped in sterile sampling bags, ensuring any adhering soil was removed. The soil from five sampling points was thoroughly mixed to create a single composite sample, with each experimental group replicated three times, resulting in nine experimental samples.

Upon collection, the specimens were immediately placed in a refrigerated sampling container and transported to the laboratory. Certain soil samples underwent air-drying and were sieved through a 40-mesh sieve for physicochemical analysis, while the remaining specimens were preserved at −80°C for the isolation of culturable microorganisms and high-throughput sequencing.

### 2.2 Soil physical and chemical property analysis

Soil pH was assessed using a Youke P901 benchtop pH meter (Shanghai Youke Instrumentation Co., Ltd., Shanghai, Chinac) with a 1:3 (w/v) water-soil suspension (Aditya et al., 2023). TP concentrations were analyzed via NaOH alkali fusion followed by molybdenum-antimony spectrophotometry (Manika and Ayele, 2022). TK content was measured using the Kjeldahl method (Hicks et al., 2022). AP was evaluated by employing the method for determining soil available phosphorus (Lin et al., 2021). AK was assessed through the methodology for determining soil available and slow-releasing potassium content (Watson et al., 2015). AN levels were determined using the alkaline diffusion technique (Yousuf and Mahmud, 2022). SOM concentration was quantified utilizing a spectrophotometer in conjunction with the SOM kit (Shanghai Lianmai Biological Engineering Co., Ltd. Shanghai, China).

### 2.3 Extraction and identification of phenolic acids in soil

The desiccated soil sample was pulverized in a ball mill and subsequently sieved through an 18-mesh filter. The processed soil was then stored in an airtight container. A quantity of 30 g of the sieved soil was placed into a conical flask, mixed with 50 mL of a 1 mol/L sodium hydroxide solution, and stirred for 4 h. The resultant mixture from the conical flask was transferred to a centrifuge tube and centrifuged at 4,000 rpm for 5 min. The pH of the supernatant was adjusted to 2.0 using 12 mol/L HCl, followed by an additional centrifugation for 10 min. The supernatant was extracted three times with 50 mL of ethyl acetate. The combined ethyl acetate layer was then moved to a round-bottom flask for rotary evaporation until dry. Subsequently, 1 mL of methanol was introduced to the flask, well-mixed until fully dissolved, and filtered through a 0.22 μm syringe filter. The resulting filtrate was then placed into a sampling vial for subsequent instrumental analysis.

Each compound—5.1 mg of coumaric acid, 4.6 mg of vanillic acid, 5.0 mg of salicylic acid, 5.4 mg of benzoic acid, 4.6 mg of bisphenol A, 4.4 mg of cinnamic acid, 4.8 mg of gallic acid, 4.6 mg of syringic acid, and 5.0 mg of ferulic acid—is accurately weighed and introduced into individual 1 mL volumetric flasks to create their respective stock solutions. For dilution purposes, combine 0.1 mL of the coumaric acid stock solution with 9.1 mL of 80% methanol; mix 0.5 mL each of the vanillic, salicylic, and benzoic acid stock solutions with 2 mL of 80% methanol, respectively; blend 0.2 mL each of the bisphenol A and cinnamic acid stock solutions with 9.8 mL of 80% methanol, respectively; and incorporate 0.5 mL each of the gallic and syringic acid stock solutions with 9.5 mL of 80% methanol, respectively. Transfer 1 mL of each diluted solution into a 10 mL test tube to formulate the mixed standard solution. Finally, add 1 mL of the mixed standard solution to a 1.5 mL volumetric flask and filter using a 0.22 μm membrane.

High-performance liquid chromatography (HPLC) was conducted with an OAA C18 column (250 mm × 4.6 mm, 5 μm) and a guard column (20 mm × 4.6 mm, 5 μm), using a detection wavelength of 280 nm. The HPLC separation utilized a mobile phase comprising 0.1% v/v formic acid in water and acetonitrile, with a column temperature of 30°C, an injection volume of 20 μL, and a flow rate of 1.0 mL/min. The elution gradient was set as follows: 5–10% acetonitrile for 0–10 min, then 10% acetonitrile for 10–20 min, increasing to 35% over 20–60 min, to 60% over 60–75 min, held at 60% for 75–85 min, decreased to 5% at 85–86 min, and maintained at 5% for 86–90 min, with a post-run of 5 min. Mixed standard samples and test filtrates of varying concentrations were analyzed using the HPLC system, followed by chromatographic data processing. Retention times of various phenolic acid metabolites served as the criteria for identification.

### 2.4 DNA extraction, PCR, and high-throughput sequencing

We extracted microbial DNA from rhizosphere soil samples of plants exhibiting three distinct health conditions, utilizing the E. Z. N. A.^®^ Soil DNA Kit (Omega Bio-tek, Norcross, GA, United States) in accordance with the manufacturer’s guidelines. The V3-V4 region of the bacterial 16S ribosomal RNA gene was amplified using the primers 341F 5′-CCTAYGGGRBGCASCAG-3′ and 806R 5′-GGACTACNNGGGTATCTAAT-3′ through PCR techniques. For the fungal 18S ribosome, the ITS1 region was amplified with the primers ITS1F 5′-CTTGGTCATTTAGAGGAAGTAA-3′ and ITS2R 5′-GCTGCGTTCTTCATCGATGC-3′. Each sample was uniquely tagged with an 8-base barcode sequence, which underwent amplification via PCR (95°C for 2 min, followed by 95°C for 30 s, 55°C for 30 s, 72°C for 30 s, culminating in a final extension at 72°C for 5 min). The PCR reactions were conducted in triplicate within a 20 μL mixture comprising 4 μL of 5 × FastPfu Buffer, 2 μL of 2.5 mM dNTPs, 0.8 μL of each primer (5 μM), 0.4 μL of FastPfu Polymerase, and 10 ng of template DNA. Subsequently, the amplicons were extracted from 2% agarose gels and purified according to the manufacturer’s instructions using the AxyPrep DNA Gel Extraction Kit (Axygen Biosciences, Union City, CA, United States).

The purified PCR products were quantified using the Qubit^®^ 3.0 system (Life Technologies, Invitrogen), and every 24 amplicons with distinct barcodes were mixed in equal proportions. This pooled DNA was then utilized to create an Illumina paired-end library, adhering to Illumina’s genomic DNA library preparation protocols. Subsequently, the amplicon library underwent paired-end sequencing (2 × 250) on an Illumina MiSeq platform (Shanghai BIOZERON Co., Ltd), following the established standard protocols. The resultant raw reads were submitted to the NCBI Sequence Read Archive (SRA) database under Accession Number: PRJNA 1170051.

### 2.5 Processing of sequencing data

Utilizing UPARSE truncation (version 7.1) with a clustering similarity threshold of 97% for operational taxonomic units (OTUs),^1^ chimeric sequences identified and eliminated using UCHIME (Stackebrandt and Goebel, 1994; Edgar, 2013). Species classification annotations were executed for each sequence via the RDP classifier (version 2.2),^2^ with a comparison threshold established at 80% (Wang et al., 2007). To retrieve the species classification information linked to each OTU, the uclust algorithm was employed for the taxonomic assessment of OTU representative sequences, and the community structure of each sample was analyzed across various taxonomic levels: domain, phylum, class, order, family, genus, and species.

The rarefaction analysisbased on Mothur v.1.21.1 (Schloss et al., 2009) was conducted to reveal the diversity indices, including the Chao, ACE, and Shannon diversity indices. The beta diversity analysis was performed using UniFrac (Lozupone et al., 2011) to compare the results of the principal component analysis (PCA) using the community ecology package, R-forge (Vegan 2.0 package was used to generate a PCA figure). Mantel tests were carried out to examine the Spearman’s rank correlation between the environmental factor and the microflora similarity using Bray-Curtis distance matrices with 999 permutations, using the vegan package in R. Multivariate analysis of variance (MANOVA) was conducted to further confirm the observed differences. The Spearman’s correlation coefficients were assessed to determine the relationships between microbiota and chemical factor such as signaling molecules. Correlation was considered significant when the absolute value of Spearman’s rank correlation coefficient (Spearman’s r) was >0.6 and statistically significant (*p* < 0.05). All statistical analysis were performed by R stats package. The R (pheatmap package) and Cytoscape^3^ were applied to visualize the relationships through correlation heatmap and network diagrams, respectively. Redundancy analysis (RDA) was employed to explore the relationship between environmental factors and microflora. One way analysis of variance (ANOVA) tests were performed to assess the statistically significant difference of diversity indices between samples. Differences were considered significant at *p* < 0.05. Venn diagrams were drawn using online tool “Draw Venn Diagram”^4^ to analyze overlapped and unique OTUs during the treatment processes. One-way permutational analysis of variance (PERMANOVA) was performed using R vegan package to assess the statistically significant effects of treatment processes on microflora.

For identification of biomarkers for Microorganisms that cause soil borne diseases, LEfSe (linear discriminant analysis effect size) analysis was done (Segata et al., 2011). Kruskal-Wallis sum-rank test was performed to examine the changes and dissimilarities among classes followed by LDA analysis to determine the size effect of each distinctively abundant taxa (Ijaz et al., 2018). Phylogenetic Investigation of Communities by Reconstruction of Unobserved States (PICRUSt)^5^ program based on the KyotoEncyclopedia of Genes and Genomes (KEGG) database was used to predict the functional alteration of microbiota in different samples. The OTU data obtained were used to generate BIOM files formatted as input for PICRUSt v1.1.09 with the make.biom script usable in the Mothur. OTU abundances were mapped to Greengenes OTU IDs as input to speculate about the functional alteration of microbiota.

## 3 Results

### 3.1 Soil physical and chemical properties of different levels of disease

Table 1 presents the physical and chemical characteristics of healthy rhizosphere soil (TN1), diseased rhizosphere soil (TN2), and severely diseased rhizosphere soil (TN3) from mango trees. The physical and chemical attributes of the soil—including TP, TK, AN, AP, AK, and OM—varied significantly among the three mango tree species experiencing different levels of disease. The average TP content followed the order of TN3 (1.00 g/kg) > TN2 (0.70 g/kg) > TN1 (0.55 g/kg). Similarly, the average TK content ranked as TN3 (20.17 g/kg) > TN2 (18.10 g/kg) > TN1 (15.80 g/kg). The average AN levels were 51.0 mg/kg for TN1, 139.0 mg/kg for TN2, and 155.33 mg/kg for TN3. The average AP content was highest in TN3 at 221.27 mg/kg, followed by TN2 at 60.07 mg/kg, and TN1 at 9.80 mg/kg. For average AK content, the order was again TN3 (221.27 mg/kg) > TN2 (215.50 mg/kg) > TN1 (89.05 mg/kg). The average OM concentration for TN1, TN2, and TN3 was 0.26, 0.86, and 1.23 mg/kg, respectively. All samples were classified as weakly acidic. As mango disease severity increased, the concentrations of TP, TK, AN, AP, AK, and OM in the three soil samples exhibited a trend of accumulation, with various soil physicochemical indices of TN3 reaching their peak values.

**Table 1:** Soil physical and chemical properties.

Samples	TP (g·kg^−1^)	TK (g·kg^−1^)	AN (mg·kg^−1^)	AP (mg·kg^−1^)	AK (mg·kg^−1^)	OM (mg·kg^−1^)	pH
TN1	0.55 c	15.80 c	51.0 b	9.80 c	89.05 c	0.26 c	5.95
TN2	0.70 bc	18.10 b	139.00 a	60.07 b	215.50 b	0.86 b	6.19
TN3	1.00 a	20.17 a	155.33 a	221.27 a	221.27 a	1.23 a	6.22

Different letters in the columns indicate significant differences (*P* < 0.05, *n* = 3).

### 3.2 Types and contents of phenolic acid autotoxins in soil

**Figure 2:**
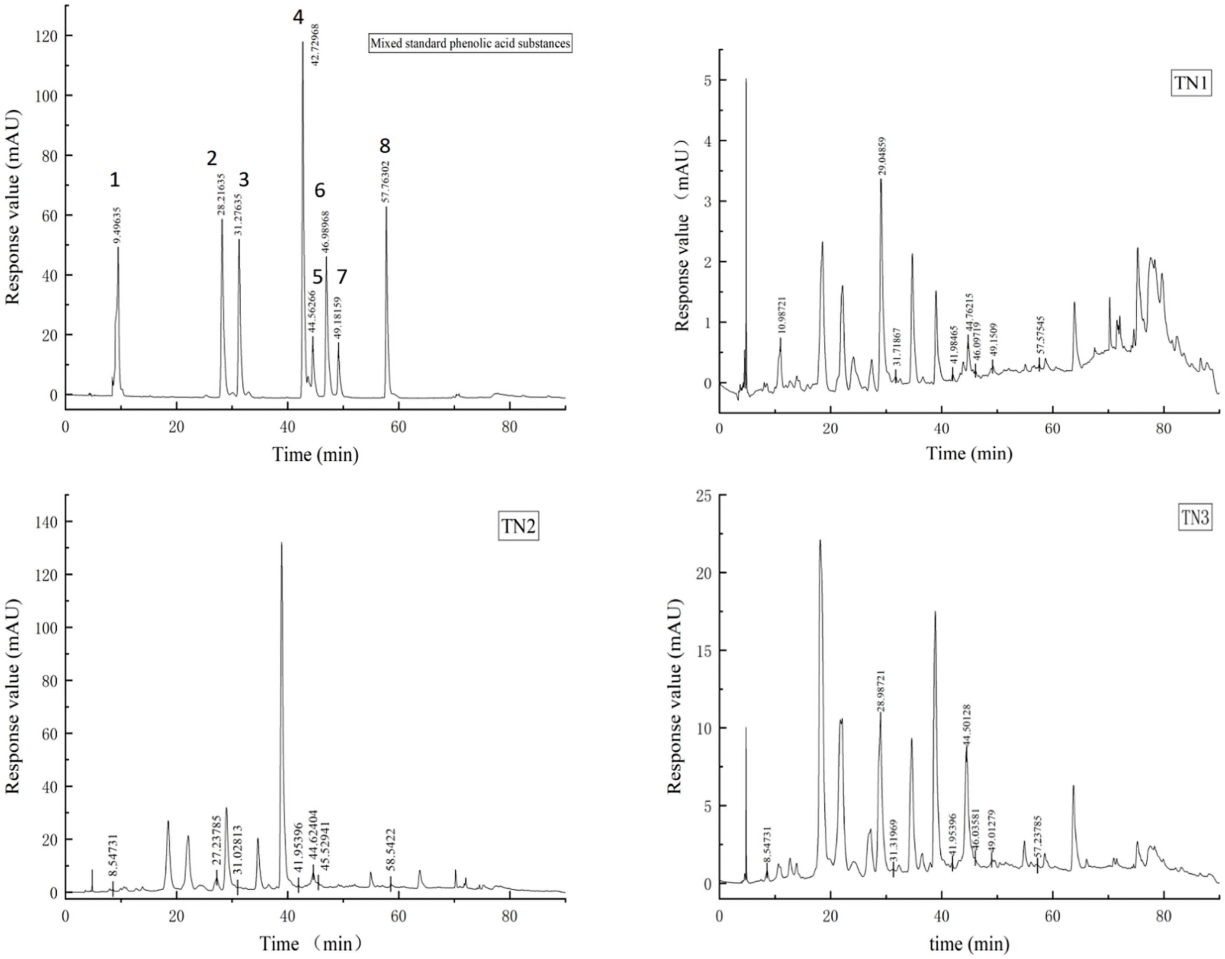
HPLC signal peak. The diagram of Mixed standard phenolic acid substances shows the signal peak of 8 phenolic acids, 1 is gallic acid, 2 is vanillic acid, 3 is syringic acid, 4 is ferulic acid, 5 is salicylic acid, 6 is benzoic acid, 7 is coumaric acid and 8 is cinnamic acid. TN1 is the healthy mango root soil, TN2 is the mango root sample with disease, and TN3 is the local mango root sample with serious disease. The number at the top of the signal peak indicates the occurrence time of phenolic acid.

Phenolic acids were extracted from the rhizosphere soil samples of healthy, diseased, and severely diseased mango plants, followed by a comparison with standard reference samples. The signal chromatogram from high-performance liquid chromatography is displayed in Figure 2. The eight phenolic acids within the mixed standard samples were distinctly separated, exhibiting clear peak profiles. The sequence of signal peaks, arranged according to polarity, is as follows: gallic acid, vanillic acid, syringic acid, ferulic acid, salicylic acid, benzoic acid, coumaric acid, and cinnamic acid. Each of the three soil samples demonstrated different levels of phenolic acid accumulation, which escalated significantly with the progression of disease severity. The concentrations of phenolic acids in the soil were quantified by measuring the peak areas, as outlined in Table 2. In comparison to TN1, the concentrations of vanillic acid, ferulic acid, salicylic acid, and coumaric acid in the soil exhibited increases of 51, 17.8, 210, and 9%, respectively; conversely, the levels of gallic acid, syringic acid, benzoic acid, and cinnamic acid experienced decreases of 68.6, 40.4, 45.2, and 45.7%, respectively. Importantly, the levels of vanillic acid first rose and then declined in TN2, a trend that is likely associated with microbial metabolic activities.

**Table 2:** Phenolic acid content in rhizosphere soil of four mango species.

Soil	Gallic acid (μg/g)	Vanillic acid (μg/g)	Syringic acid (μg/g)	Ferulic acid (μg/g)	Salicylic acid (μg/g)	Benzoic acid (μg/g)	Coumaric acid (μg/g)	Cinnamic acid (μg/g)
TN1	149.80	616.62	133.88	42.58	708.19	521.09	30.80	59.39
TN2	19.63	2205.12	34.07	17.50	1275.12	194.76	25.55	19.10
TN3	47.04	931.12	79.79	50.15	2195.37	285.73	33.60	32.23

### 3.3 OTU analysis of PCR-amplified 16S rRNA/ITS gene and ITS sequence

**Figure 3:**
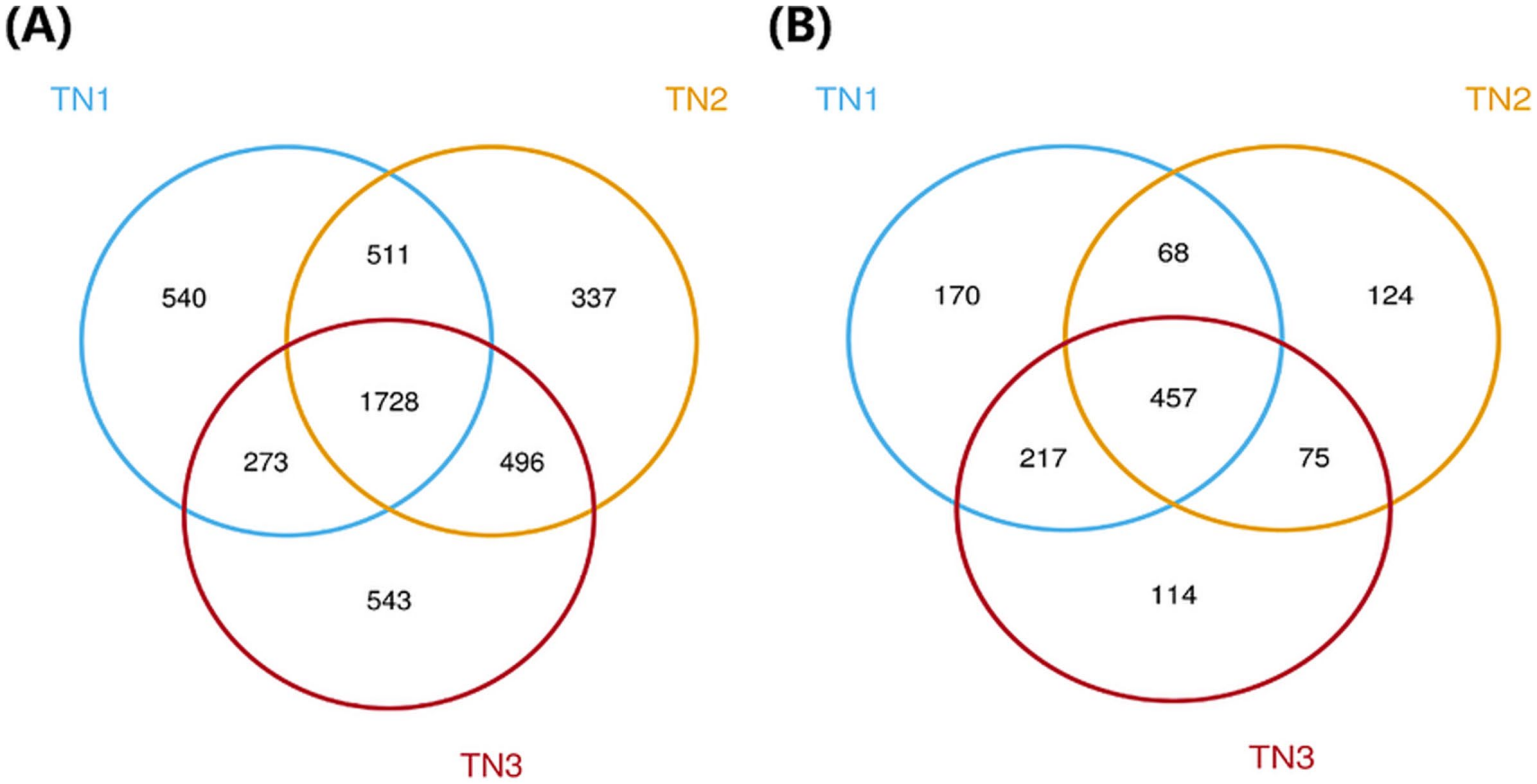
Venn diagram of bacterial **(A)** and fungal **(B)** OUTs.The different colors represent the various soil samples: blue for TN1, yellow for TN2, and red for TN3. Collectively, the three types of samples in the bacterial community have 1,728 OTUs, while the three types of samples in the fungal community have 457 OTUs.

High-throughput sequencing analysis was performed on nine samples from the mango root zone. After processing steps including splicing, decontamination, and chimera removal from the raw data, we obtained a total of 451,602 raw 16S rRNA sequences, with an average of 37,634 sequences per sample (ranging from 33,355 to 41,523), and 464,137 raw ITS sequences, averaging 38,678 sequences per sample (ranging from 34,162 to 41,887). The high-quality 16S rRNA sequences ranged from 410 to 414 bp, while the ITS sequences ranged from 235 to 267 bp. Clustering of non-redundant sequences, excluding singletons, at 97% similarity resulted in the identification of 4,725 bacterial operational taxonomic units (OTUs) and 1,756 fungal OTUs. As shown in the Venn diagram (Figure 3), bacterial OTUs were more abundant than fungal ones. The annotation ratio chart can be found in the supplementary materials (Supplementary Figures 1, 4). The category accumulation curve graph can be found in the supplementary materials (Supplementary Figures 2, 5).

### 3.4 Changes in rhizosphere microbial community diversity

**Figure 4:**
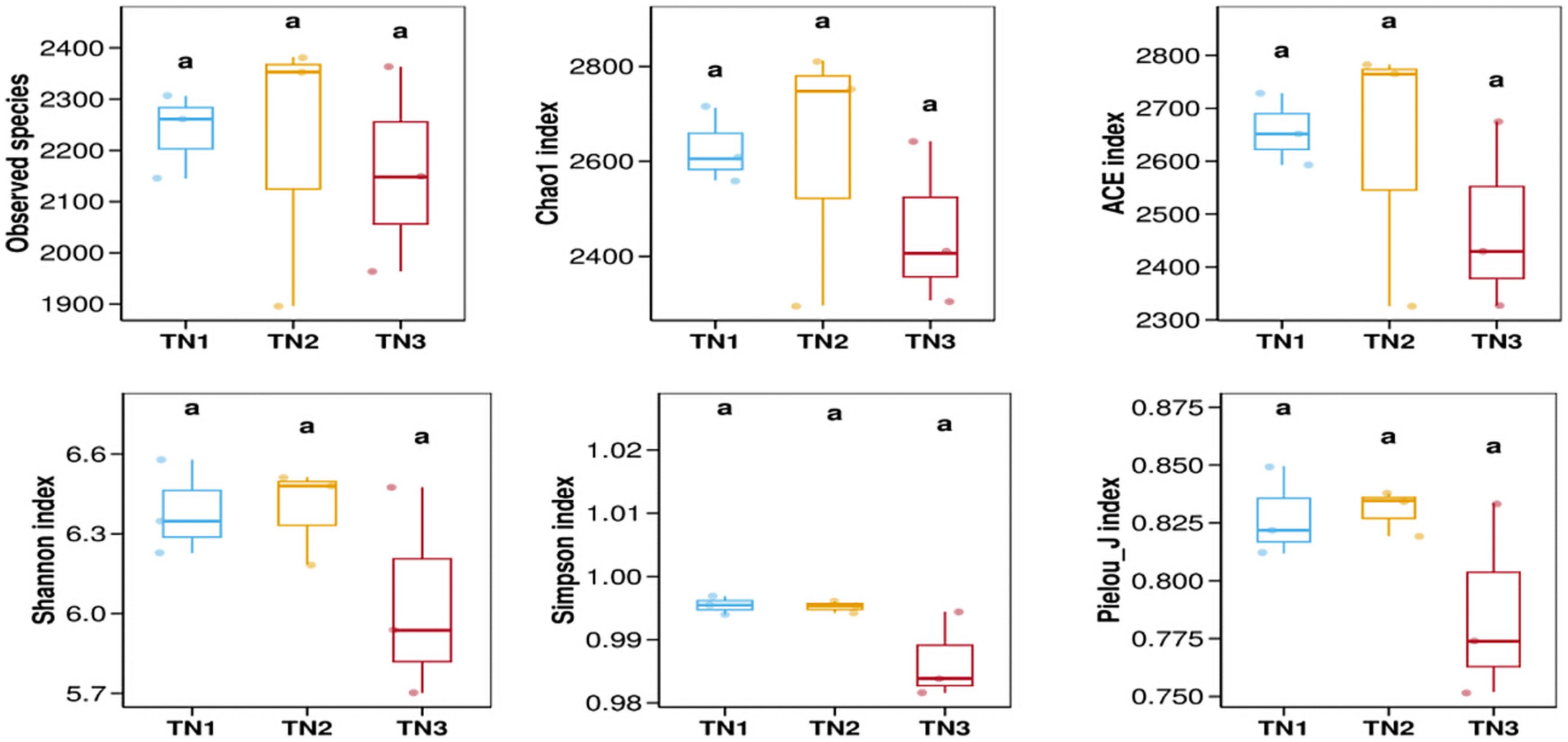
Bacterial 16 s RNA *α* diversity index. Alpha diversity reflects the richness and diversity of microbial communities within a sample. Alpha diversity indices were calculated using QIIME software, including Observed species, Chao1, ACE, Shannon, Simpson, and Pielou J. Observed species, Chao1, and ACE are richness indices; Shannon and Simpson are diversity indices; and Pielou J is an evenness index. Different letters represent significant differences (*P* < 0.05).

**Figure 5:**
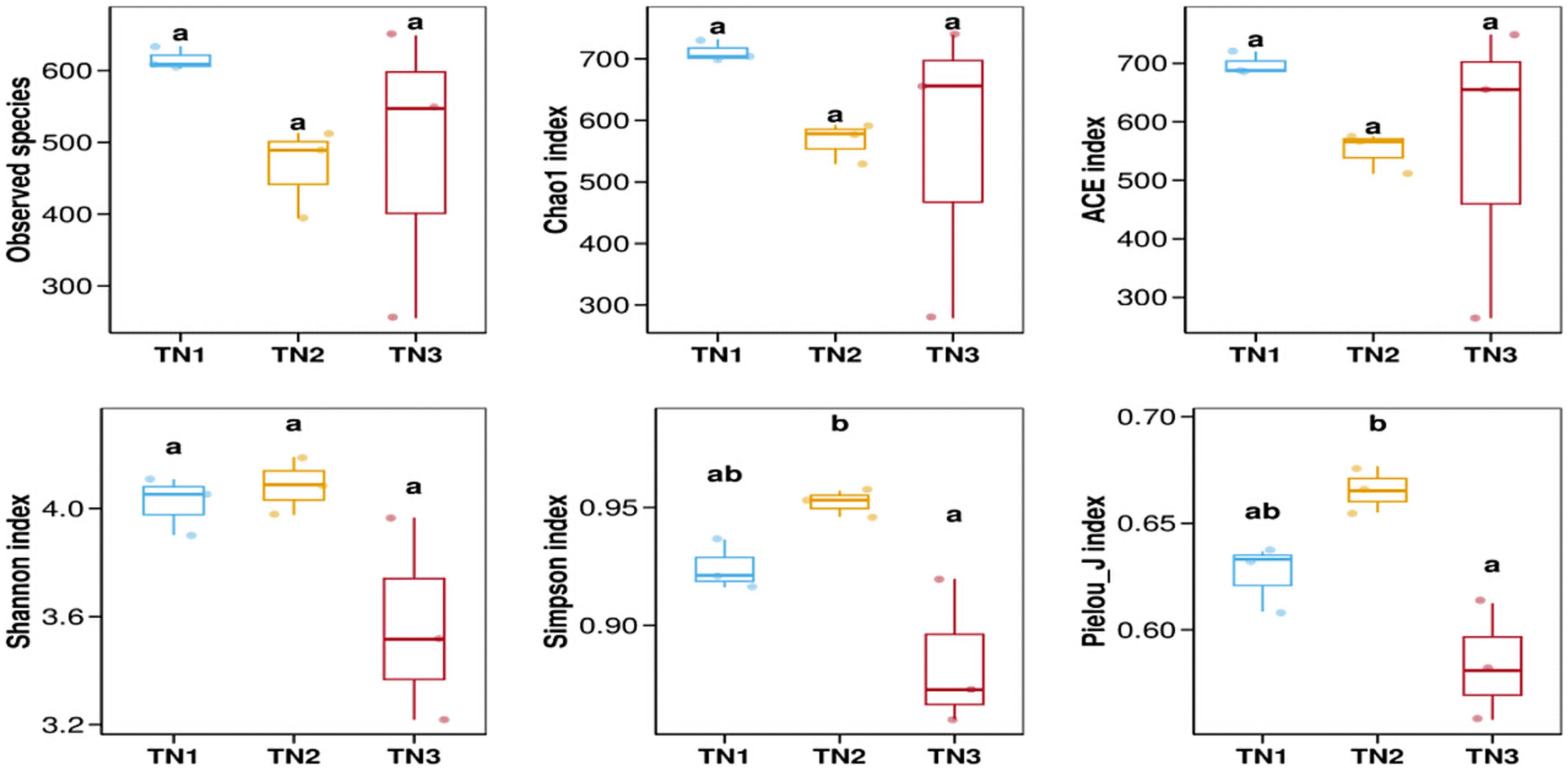
Fungal ITS α diversity index.

The variation in soil microbial communities across distinct ecological niches in both healthy mango plants and those affected by soil-borne pathogens is depicted in Figure 4. Assessment using the Observed species, Chao1, ACE, Shannon, Simpson, and Pielou_J indices reveals that the bacterial community (16S rRNA) demonstrates the highest *α* diversity index in the diseased group (TN2), while the severe disease group (TN3) shows the lowest α diversity index, but the difference between the three groups is not significant. These findings indicate a trend in rhizosphere microbial diversity in response to soil borne diseases in mangoes, marked by an initial increase followed by a pronounced decline. Notably, the fungal community (ITS) shows an α diversity index that trends inversely to that of the bacterial community, indicating a rise in diversity concurrent with a decline in bacterial diversity (Figure 5). In the TN3 group, the Shannon, Simpson, and Pielou_J indices exhibit significant deviations from those observed in other groups. Statistical analyses indicate notable differences in the α diversity indices among various sample types.

**Figure 6:**
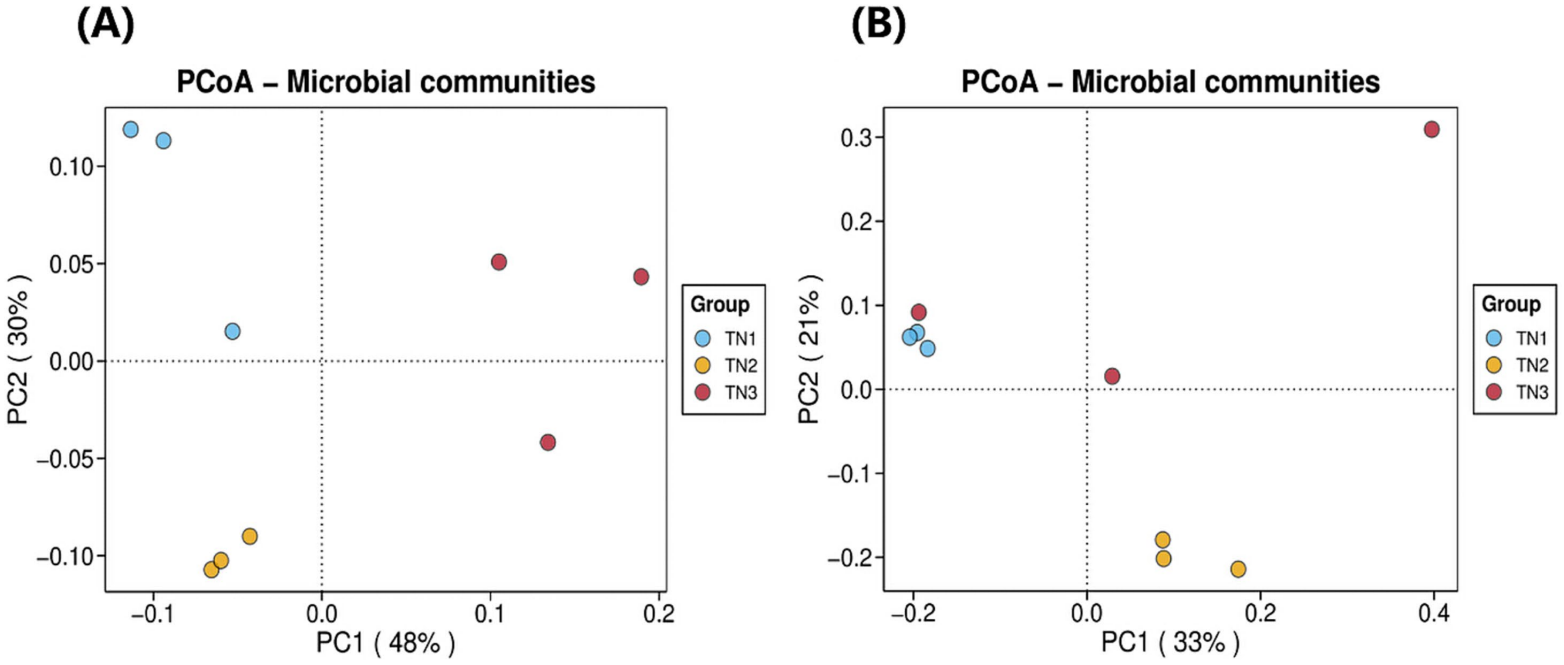
Bacterial **(A)** and fungal **(B)** PCoA analysis result chart. The horizontal coordinate indicates the first principal component, and the percentage shows its contribution to the sample variance. The vertical coordinate shows the second principal component, and the percentage shows its contribution to the sample variance. Each point on the graph represents a sample, and samples in the same group are shown in the same color.

As illustrated in Figure 6, we analyzed the composition of rhizosphere soil bacterial communities in samples TN1, TN2, and TN3 through principal coordinate analysis (PCoA). These two principal coordinates accounted for 78% of the variance in bacterial community structure across all samples, where PC1 accounted for 48% and PC2 for 30%. The bacterial community structure of the healthy group (TN1) exhibited significant differences when compared to the diseased groups (TN2 and TN3), indicating substantial alterations in the bacterial community following the occurrence of soil borne diseases in mango. Additionally, the rhizosphere soil fungal communities of the three samples were evaluated using PCoA. The two principal components explained 54% of the variation in fungal community structure, with PC1 explaining 33% and PC2 21%. Notably, samples TN2 displayed significant differences relative to the other samples, while certain TN1 samples exhibited similarities to both TN3 samples. It is particularly noteworthy that TN3 samples demonstrated significant deviations on the PC1 axis, suggesting that soil borne diseases substantially influence the community structure of rhizosphere fungi, leading to marked differences.

### 3.5 Composition of bacterial/fungal communities in the rhizosphere of mango soil

**Figure 7:**
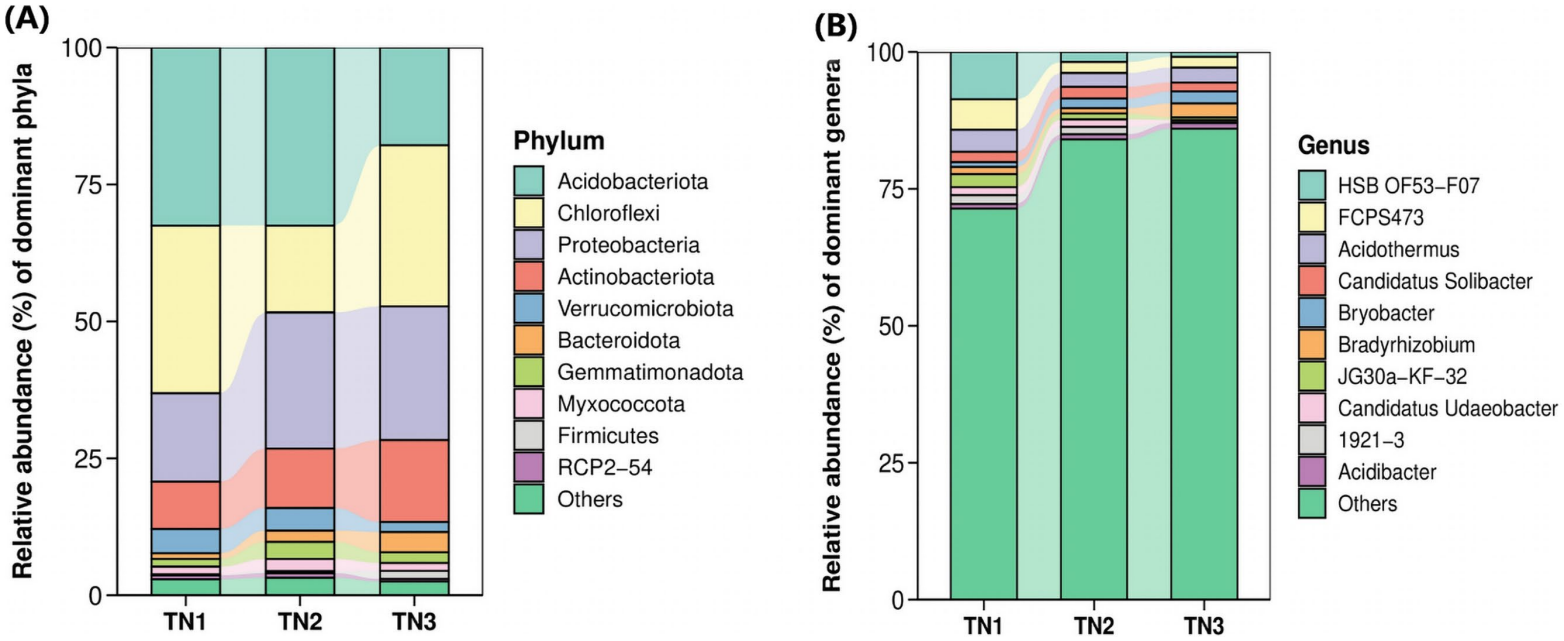
Horizontal abundance maps at phyla level **(A)** and genus level **(B)** of bacterial classification. Each bar represents a sample type that has been integrated with the same sample type (n = 3). The color blocks represent different species. The height of each color block in the stacked bar graph represents the relative abundance of that species in the corresponding sample. Color blocks of the same species in different samples are connected by a curve of the same background color, which makes it convenient to observe how the relative abundance of each species changes.

High-throughput sequencing revealed the presence of 375 genera and 821 species across 35 phyla, 84 classes, 182 orders, and 236 families within bacterial communities derived from soil samples. The 10 most dominant bacterial phyla (Figure 7A), namely Acidobacteria, Chloroflexi, Proteobacteria, Actinobacteria, Verrucomicrobiota, Bacteroidota, Gemmatimonadota, Myxococcota, RCP2-54, and WPS-2, collectively comprised 96.53–97.96% of the total microbial abundance in the rhizosphere soil of mango trees. In particular, Acidobacteria exhibited the highest abundance in TN1 and TN2 soils, accounting for 32.49%, but demonstrated a significant decline in TN3. The relative abundances of Proteobacteria, Actinobacteria, and Bacteroidota showed a progressive increase, whereas Acidobacteria and Verrucomicrobiota presented a downward trend. Notably, Chloroflexi abundance first decreased, then increased, showing a unique trend.

To investigate the variations in the abundance and composition of soil bacteria within the rhizospheres of mango trees affected by soil-borne pathogens, analyses were performed at the genus level (Figure 7B). The six predominant bacterial genera observed across all treatment groups included HSB OF53−F07, Acidothermus, FCPS473, Candidatus Solibacter, Bradyrhizobium, and Bryobacter. Importantly, as the disease progressed, the populations of HSB OF53 − F07, Acidothermus, and FCPS473 experienced significant declines (from 8.63, 4.04, and 5.57% in TN1 to 0.91, 2.70, and 1.97% in TN3), whereas Bradyrhizobium (from 1.29 to 2.52%) and Bryobacter (from 0.88 to 2.20%) exhibited increases.

**Figure 8:**
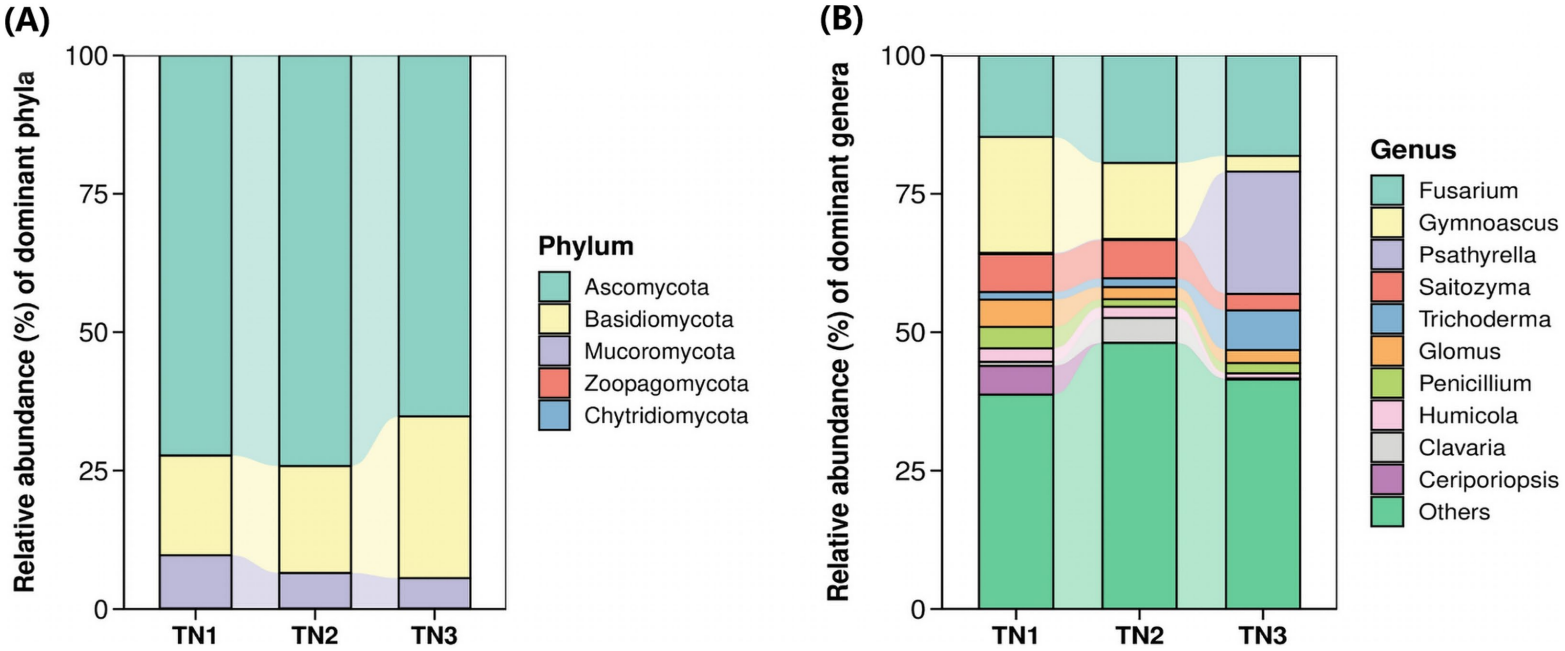
Horizontal abundance maps at phyla level **(A)** and genus level **(B)** of fungal classification.

The operational taxonomic units (OTUs) derived from soil samples were categorized, revealing the presence of 1 kingdom, 5 phyla, 26 classes, 96 orders, 228 families, 505 genera, and 898 species of fungi. At the phylum level (Figure 8A), the predominant lineages identified included Ascomycota, Basidiomycota, Mucoromycota, Chytridiomycota, and Zoopagomycota, with the first three phyla accounting for over 99.85% of the overall fungal community within the mango root zone soil. Notably, Ascomycota exhibited the highest abundance in the TN2 plot at 74.18%, while Basidiomycota reached its maximum in TN3 at 29.21%. As disease severity escalated, the proportion of Basidiomycota increased from 17.99 to 29.21%. In contrast, the levels of Ascomycota and Mucoromycota declined from 72.28 to 65.21% and from 9.62 to 5.46%, respectively. Fusarium, Gymnoascus, Psathyrella, Saitozyma, and Trichoderma represent the five most prevalent genera at the genus level (Figure 8B). As the disease advances, the populations of Fusarium, Psathyrella, and Trichoderma in the soil exhibit a significant increase, while there is a notable decline in Gymnoascus and Saitozyma populations. Notably, the relative abundance of Psathyrella is very low in samples TN1 and TN2, yet it surges in TN3, possibly in relation to mango diseases. The cumulative abundance map of the samples at the door level can be found in Supplementary Figures 3, 6).

### 3.6 The influence of environmental factors on bacterial/fungal communities

**Figure 9:**
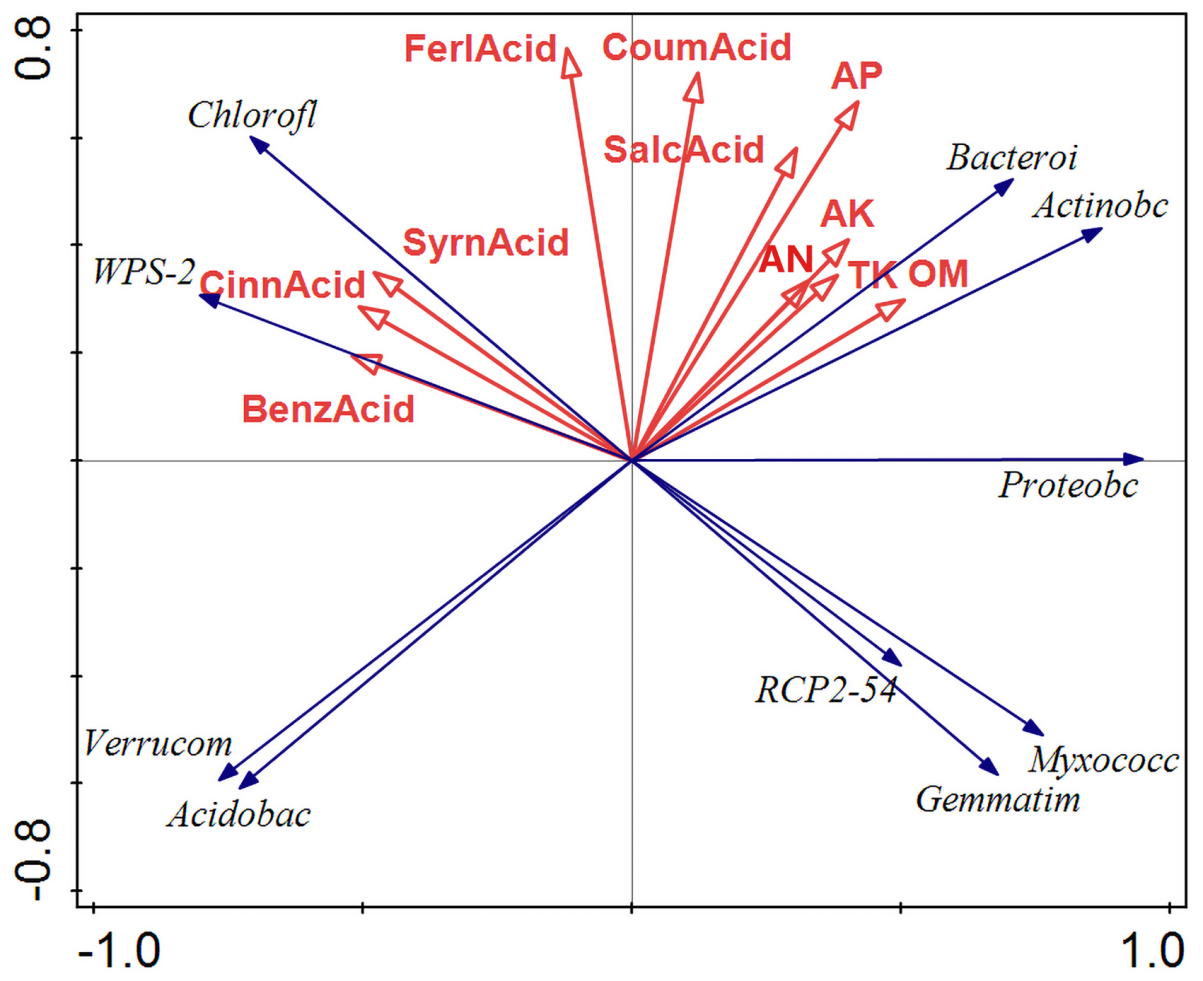
RDA results of bacterial redundancy analysis. RDA analysis was mainly used to reflect the relationship between the flora and environmental factors. The red arrows in the picture represent different environmental factors, and the blue arrows represent different bacterial communities. The abbreviations represent different meanings: Acidobac, Acidobacteria phylum; Verrucom, Verrucomicrobiota phylum; Chlorofl, Chloroflexi phylum; Bacteroi, Bacteroidota phylum; Actinobc, Actinobacteria phylum; Proteobc, Proteobacteria phylum; and Myxococc, Myxococcota phylum; Cemmatim, Cemmatimonadota; BenzAcid, benzoic acid; CinnAcid, cinnamic acid; SyrnAcid, syringic acid; SalcAcid, salicylic acid; FerlAcid, ferulic acid; CoumAcid, coumaric acid; AP, effective phosphorus; AN, alkaline hydrolysis nitrogen; AK, available potassium; TK, total potassium; OM, organic matter.

**Figure 10:**
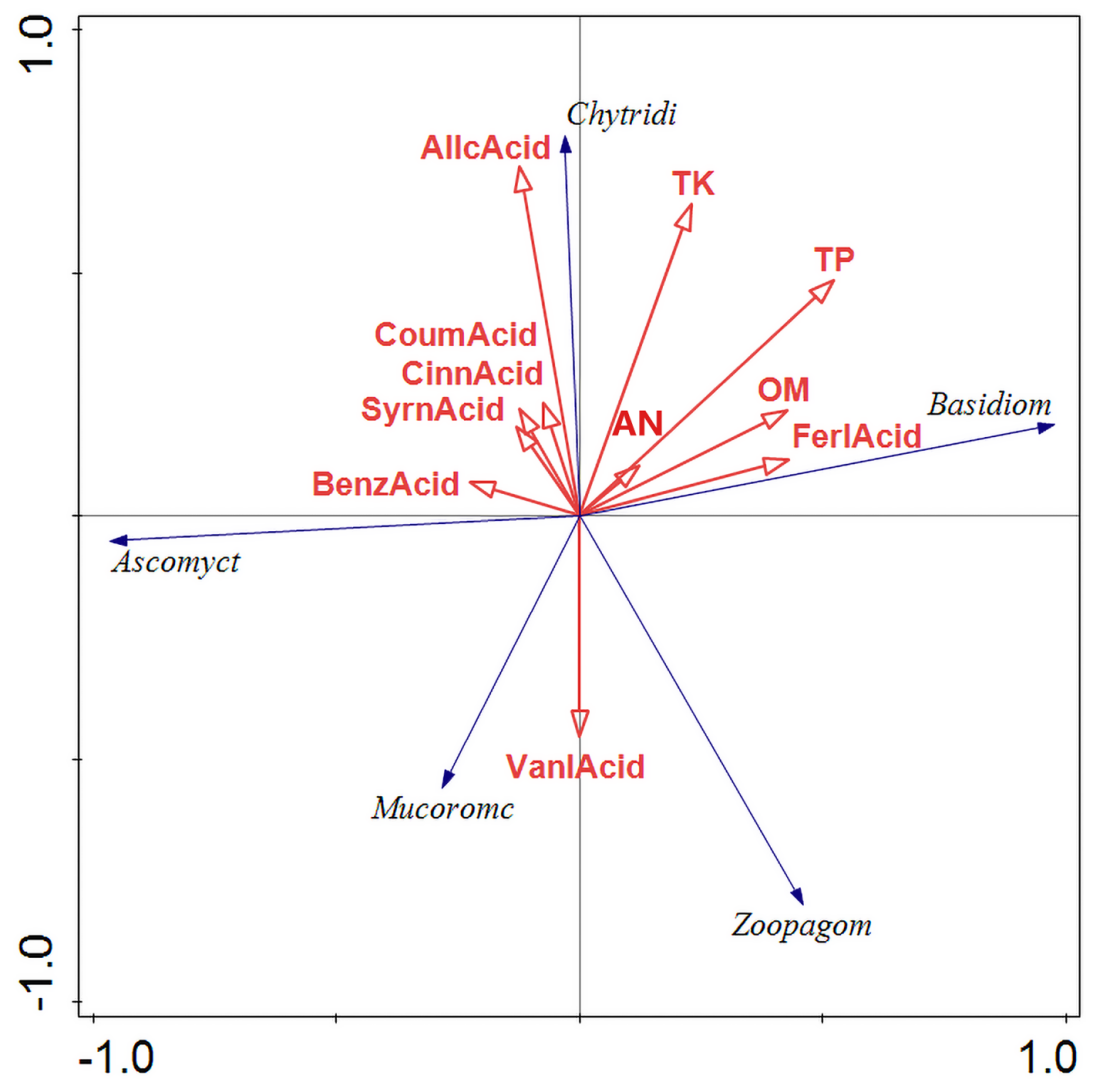
RDA results of fungus redundancy analysis. RDA analysis was mainly used to reflect the relationship between the flora and environmental factors. The red arrows in the picture represent different environmental factors and the blue arrows represent different fungal communities. The abbreviations represent different meanings: Basidiom, Basidiomycola phylum; Mucoromc, Mucoromycola phylum; Ascomyct, Ascomycola phylum; Chytridi, Chytridiomycola phylum; BenzAcid, benzoic acid; CinnAcid, cinnamic acid; SyrnAcid, syringic acid; SalcAcid, salicylic acid; FerlAcid, ferulic acid; CoumAcid, coumaric acid AllcAcid, salicylic acid; AP, acid-soluble phosphorus; AN, alkaline hydrolysis nitrogen; AK, available potassium; TK, total potassium; OM, organic matter; TP, total phosphorus.

The results of the bacterial redundancy analysis (RDA) are presented at the classification gate level (Figure 9), indicating that acid available phosphorus (AP) is the predominant environmental factor influencing bacterial communities, contributing to 22.4% of the variations observed. In a similar vein, total phosphorus (TP) significantly affects fungal communities (Figure 10), accounting for 22.4% of their variations. AP demonstrates a positive correlation with Bacteroides and Actinobacillus, while exhibiting a negative correlation with Verrucomicrobia and Acidobacillus. Meanwhile, TP shows a positive relationship with Basidiomycetes and a negative correlation with Mucoromycetes.

**Figure 11:**
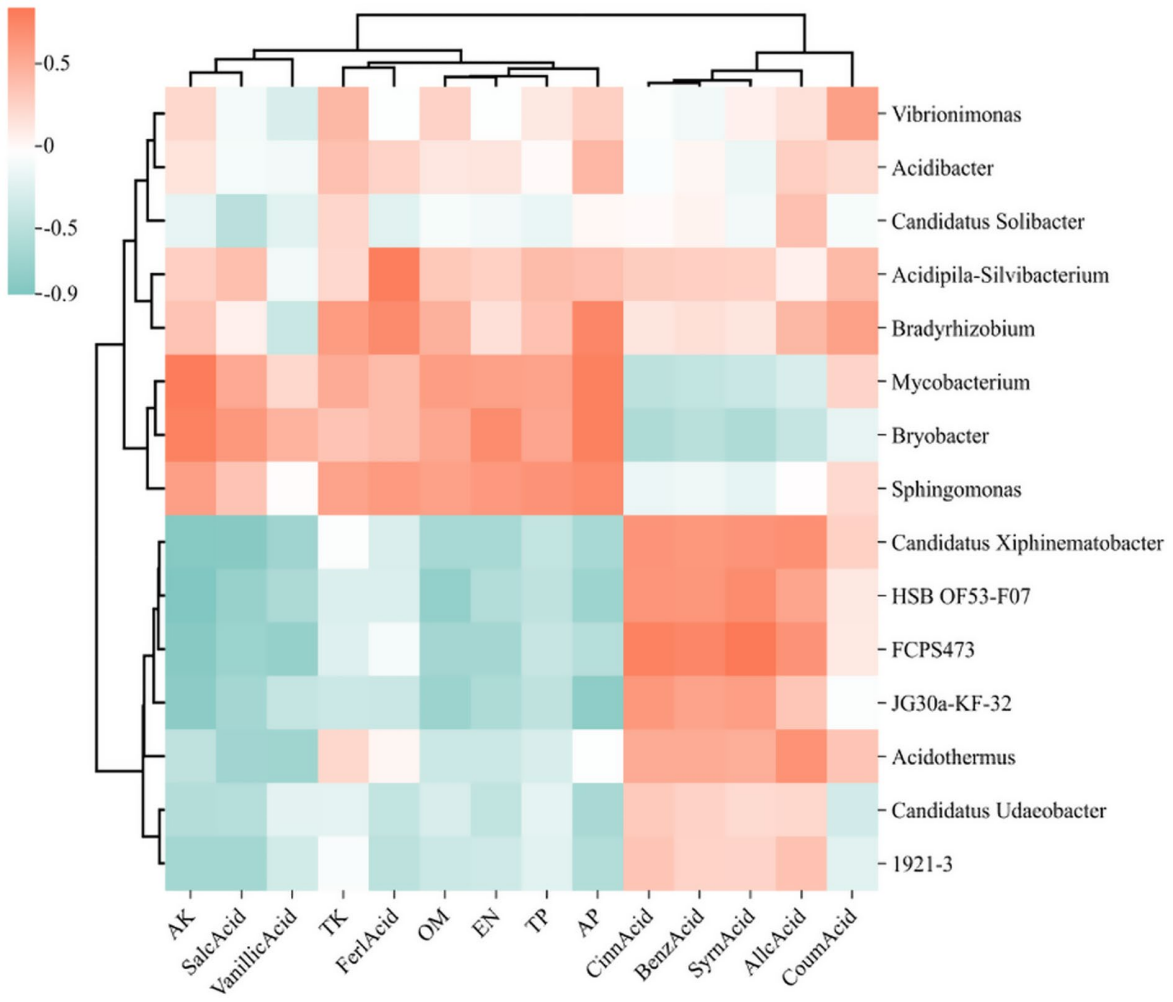
Heat map of the relationship between the 15 bacteria genera with the highest relative abundance, soil physicochemical properties, and phenolic acids. The relative abundance of each sample was calculated based on the percentage of total effective sequences classified by the RDP classifier and compared with the Silva and UNITE gene databases.

**Figure 12:**
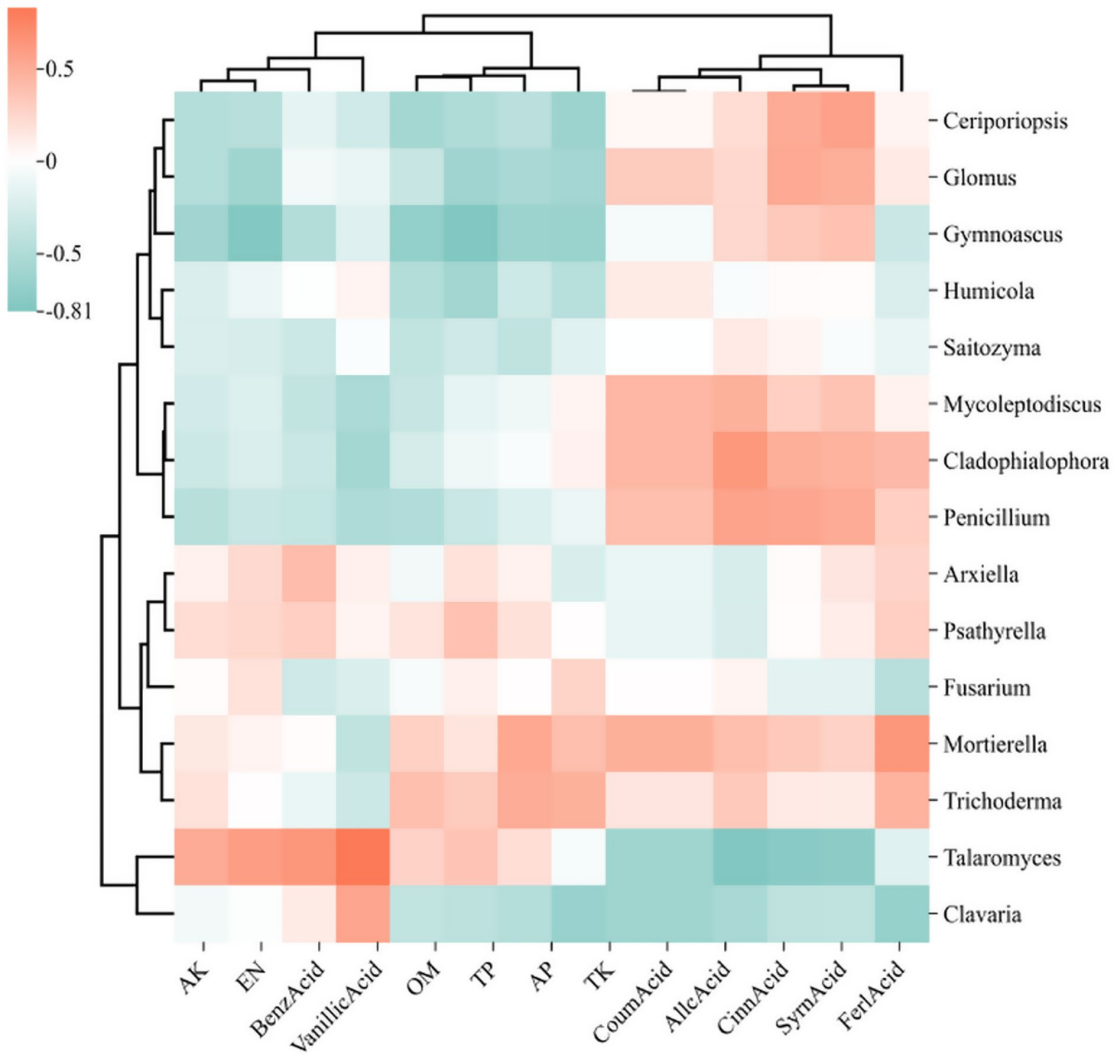
Heat map of the relationship between the 15 fungi genera with the highest relative abundance, soil physicochemical properties, and phenolic acids. The relative abundance of each sample was calculated based on the percentage of total effective sequences classified by the RDP classifier and compared with the Silva and UNITE gene databases.

The correlation heatmap illustrates the relationships among microbial genera, soil physicochemical properties, and root exudate, where deeper red hues signify stronger positive correlations and deeper green hues indicate stronger negative correlations. In the heatmap focusing on the top 15 bacterial genera (Figure 11), alkali hydrolyzable nitrogen (AN), total phosphorus (TP), and acid-extractable phosphorus (AP) demonstrated positive correlations with Sphingomonas, Bryobacter, and Mycobacterium, while exhibiting negative correlations with 1921–2, HSB OF53-F07, and JG30a-KF-32. Conversely, autotoxic compounds such as cinnamic acid, benzoic acid, and syringic acid were found to have significant positive correlations with Candidatus Xiphinematobacter, HSB OF53-F07, and FCPS473. In the fungal genera heatmap (Figure 12), available potassium (AK) and alkaline hydrolyzable nitrogen (AN) revealed strong positive correlations with Talaromyces. At the same time, autotoxic compounds like coumaric acid, gallic acid, and cinnamic acid exhibited predominantly strong positive correlations with Penicillium, Cladophialophora, and Mycoleptodiscus.

### 3.7 Soil microbial functioning potential in rhizosphere

**Figure 13:**
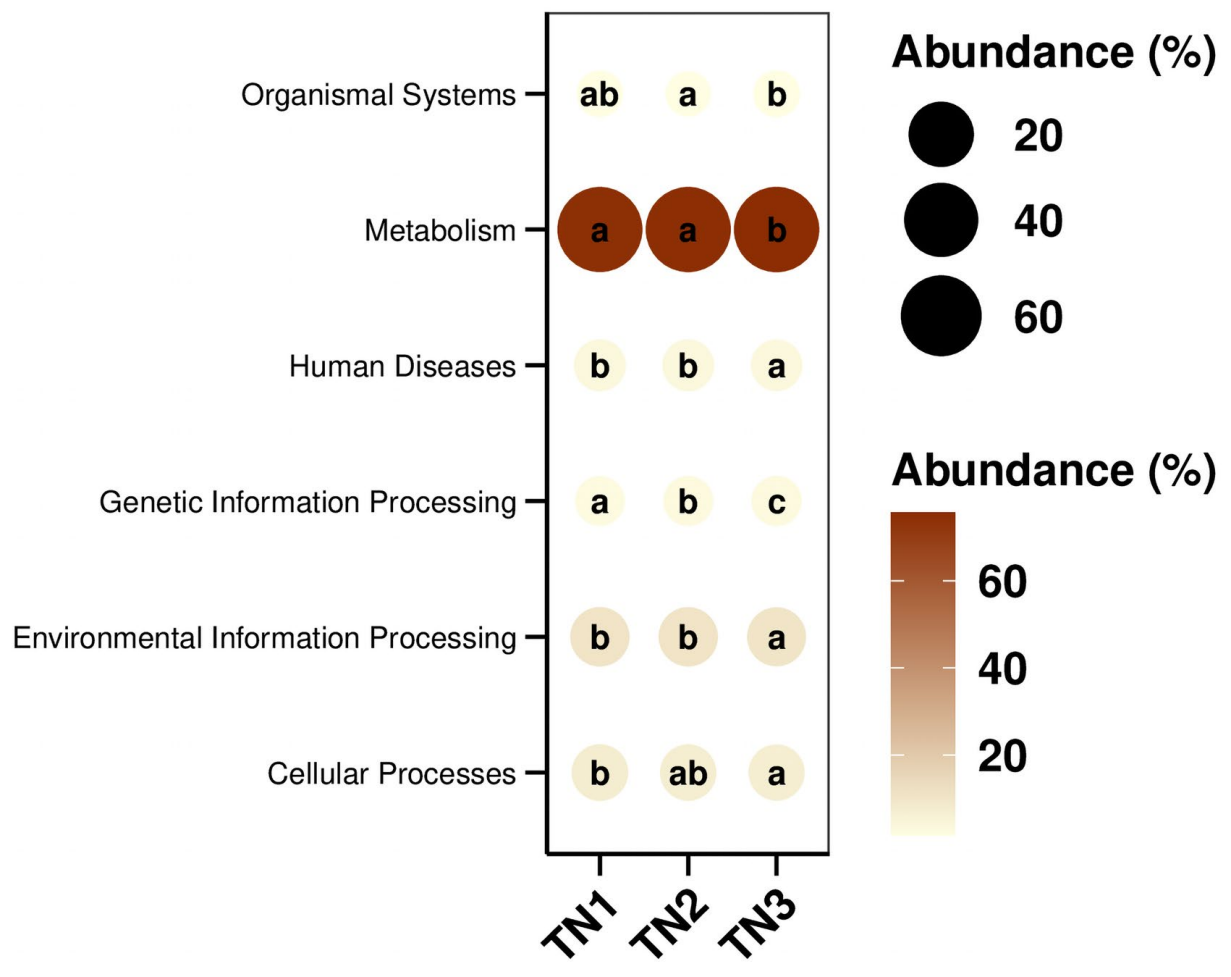
KEGG path group difference bubble map. The size and color of the dots in the figure correspond to the average relative abundance of the corresponding pathway in the respective group. The lowercase letters in the circles represent the results of the significance test for the difference in pathway abundance between groups. Letters are different when *p*-value is less than 0.05.

**Figure 14:**
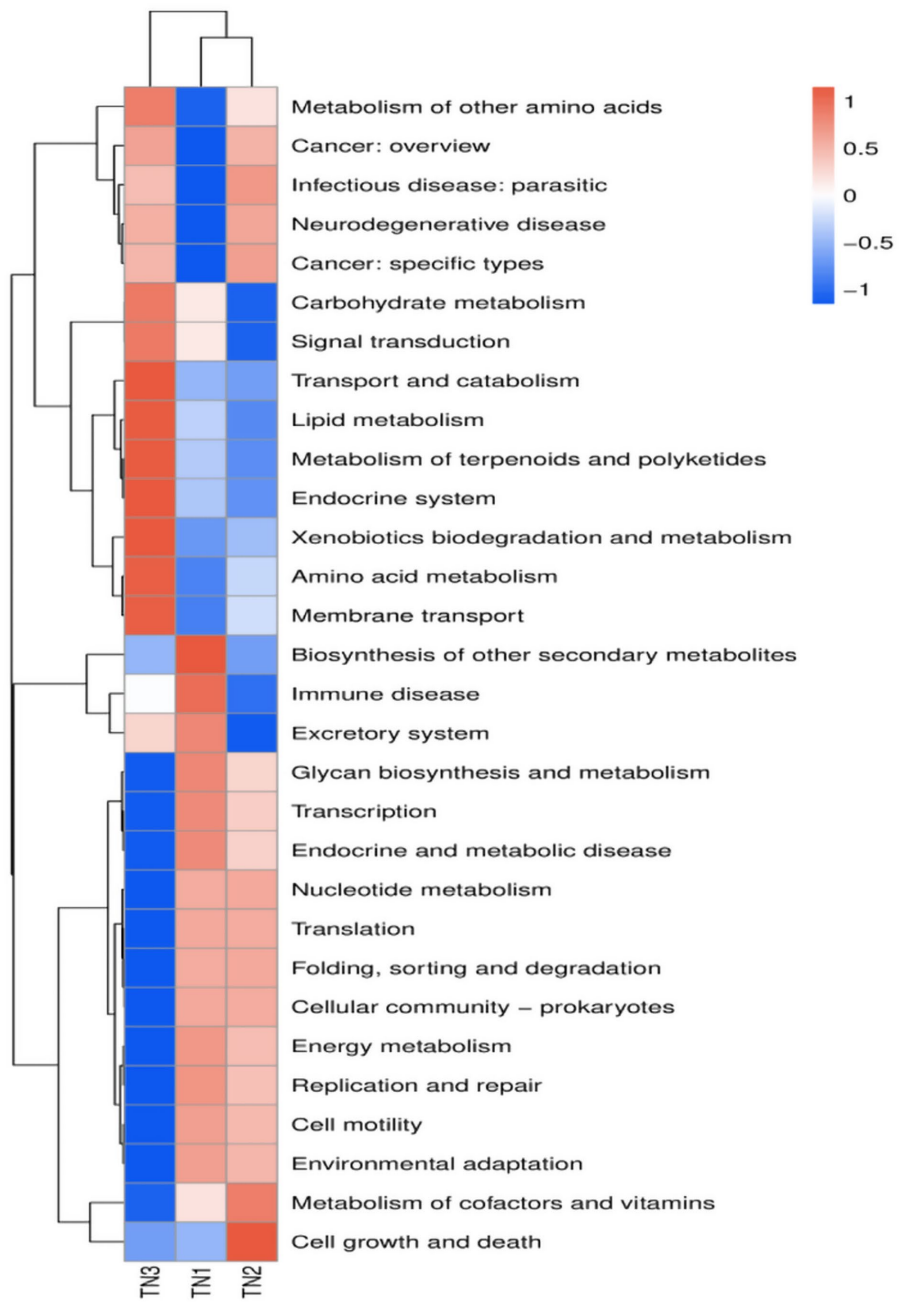
PICRUSt2: Predicting abundance information in heatmaps. Based on the function annotations and abundance information of the samples in the database, the 35 most abundant functions and their abundance in each sample were selected to create heat maps. The functions were then clustered based on functional differences.

PICRUSt2 was implemented to predict the functioning potential of soil bacterial communities in Mango. The microbial functions identified in the samples in this study are consistent with 6 primary metabolic pathways (Level 1) and more than 30 secondary metabolic pathways (Level 2) in the Kyoto Encyclopedia of Genes and Genomes (KEGG). According to the bubble map of KEGG primary metabolic pathway differences (Figure 13), there were significant differences in the function of rhizosphere soil bacteria between healthy and diseased mango, especially in gene information processing, metabolism and organism system. According to Figure 14, it can be observed that at the level of secondary metabolism, healthy and diseased plant rhizosphere microorganisms play different biological roles, indicating that soil borne diseases affect the community function of rhizosphere microorganisms.

## 4 Discussion

### 4.1 Effects of soil borne diseases on physical and chemical properties of rhizosphere soil

The physical and chemical characteristics of soil are crucial indicators of its quality and have a significant impact on plant growth. The physical and chemical properties of mango rhizosphere soil with different diseases were examined, most of the physical and chemical parameters of TN3 showed marked differences when compared to TN2 and TN1, with a notable trend of increase as the severity of disease escalated. This suggests that following the onset of soil borne diseases, the roots’ capacity to absorb external nutrients diminishes, resulting in the accumulation of nutrients within the soil. Long-term continuous cropping of soil will affect soil properties, it was found that total N, total K and available K in soil increased with the increase of banana continuous cropping years (Zhong et al., 2014), which aligns with the findings of this research. Nitrogen, phosphorus, potassium, and organic matter are vital for plant development, but excessive amounts can be detrimental. When nutrient absorption is lower than nutrient release, it leads to nutrient accumulation around the roots, creating favorable conditions for pathogen proliferation. Research has indicated that nutrient inputs can influence soil fungal communities that are essential for ecosystem functioning, where nitrogen and phosphorus enhance the prevalence of fungal pathogens while suppressing beneficial symbiotic organisms (Lekberg et al., 2021). The results indicate a correlation between the physical and chemical soil properties and soil borne diseases. Therefore, the next step of research is to finally describe and clarify the relationship between nutrients and microbial community.

### 4.2 Changes in root exudates caused by soil borne diseases

Root exudates from plants may constitute as much as 25% of the carbon assimilated through photosynthesis, which includes organic acids, carbohydrates, and various other compounds (Zhang and Mason, 2022). Plants enhance their environmental adaptability by modifying this composition of root exudates and altering the makeup of soil microorganisms (Zhalnina et al., 2018). Certain exudates function as signaling molecules, influencing soil characteristics and facilitating nutrient uptake and utilization (Vives-Peris et al., 2020). Phenolic acids can promote pathogens at low concentrations while exhibiting inhibitory effects at elevated concentrations, depending on the plant’s growth condition (Li et al., 2018). In order to verify this dynamic change, the content of phenolic acids in soil was detected by HPLC in this study. Eight phenolic acids were identified from mango root soil, among which vanillic acid, salicylic acid and benzoic acid were the most common (Table 2). Notably, vanillic acid levels were markedly higher in the disease-affected group compared to the healthy counterparts, peaking at TN2 before declining, which may indicate utilization by microorganisms. Additionally, some research suggests that vanillic acid plays a role in bolstering root resistance against pathogens (Corrales-Sánchez et al., 2022). This implies that the observed increase in vanillic acid concentration in this study may signify a mechanism of plant disease resistance. Salicylic acid, a common phenolic compound in plants, is known for regulating growth and developmental processes (Li et al., 2022). Furthermore, it serves as a principal defense hormone for plants confronted with diverse pathogens and abiotic stresses (Spoel and Dong, 2024). The present study revealed that as the impact of soil borne diseases on mango trees intensified, the accumulation of salicylic acid secreted by roots escalated, reaching concentrations of 2,195.37 μg per gram of soil in TN3. The levels of phenolic acids in root soil can influence the structure of soil microbial communities; In general, there were some differences in the types and contents of mango root exudates with different disease degrees, which was related to the mechanism of plant disease prevention. However, the type and content of secretions can play a disease-resistant effect still need to be further explored.

### 4.3 Effects of soil borne diseases on soil microbial community structure

Our findings elucidate the soil microbiome’s reaction to soil-borne pathogens and the resultant alterations in microbiome dynamics. An analysis through high-throughput sequencing indicated that total bacterial biomass was notably higher in the rhizosphere of healthy plants compared to diseased specimens, whereas the opposite was observed for total fungal biomass. We employed various metrics, including the Observed Species Index, Chao1 Index, ACE Index, Shannon Index, Simpson Index, and Pielou_J Index, to assess the richness of microbial community structure. The *α*-diversity metrics for TN1 and TN2 outperformed those of TN3, indicating greater bacterial diversity. Consistent results regarding total bacterial populations were noted in both healthy and infected cotton plants (Wei et al., 2021). Microorganisms serve as crucial indicators of soil health, particularly concerning disease management and pollution mitigation (Wu et al., 2023). Enhanced microbial diversity is linked to improved community resilience (Ma et al., 2022). Some research indicates that root rot may elevate the populations of fungal pathogens (Solís-García et al., 2021), and our study corroborated that the α-diversity index for fungi exhibited more pronounced changes in TN3. The manifestation of mango root rot appears to correlate with an increase in rhizosphere fungal abundance. Consequently, it can be inferred that the microbial communities in TN1 and TN2 demonstrate a heightened resistance to disease and possess a greater capacity to inhibit pathogen proliferation compared to TN3.

The investigation into the assembled rhizosphere microbial community revealed the establishment of a core microbiome throughout the mango growth cycle, encompassing a variety of bacterial and fungal phyla. Key constituents of this community include Acidobacteria, Actinobacteria, Chloroflexi, and Proteobacteria. Remarkably, Acidobacteria constituted an average of 26.61% of the community composition observed in this study (Figure 6). Despite the currently limited research on Acidobacteria, these bacteria are integral to ecosystem functions, as they can exploit a range of carbon sources and partake in the decomposition of sugars (Kielak et al., 2016); Furthermore, Acidobacteria are capable of utilizing both inorganic and organic nitrogen sources, with some species within this phylum demonstrating the ability to reduce nitrate (Eichorst et al., 2018). Actinobacteria, representing an average of 11.62% of the microbial population in the analyzed samples, play a crucial role as well. They not only synthesize antibiotics and lysozymes (Selim et al., 2021), but also function as biocontrol agents that inhibit soil-borne pathogens (Edelvio et al., 2018). At the genus level, the population of actinomycetes initially declined before subsequently increasing, suggesting that the enrichment of nutrient elements in the soil has facilitated the proliferation of these microorganisms. Concurrently, the phenolic acids released by the roots likely led to the preferential utilization of these organisms. All these dynamics are intricately linked to the disease resistance mechanisms of plants.

In the fungal communities examined in this research, Ascomycota, Basidiomycota, and Mucoromycota were recognized as the predominant taxa. Ascomycetes and Basidiomycetes are widely found in soil environments, showcasing an extensive array of species diversity. These groups play a crucial role in soil ecosystems by decomposing organic materials, forming mycorrhizal partnerships with vascular plants, and aiding in nutrient cycling. This symbiotic relationship not only enhances plant nutrient uptake but also fosters ecological equilibrium and boosts agricultural yields. Nevertheless, certain Ascomycete taxa may serve as pathogens impacting both plants and animals, potentially undermining agricultural productivity and ecological integrity (Miyauchi et al., 2020). In this investigation, these fungal taxa represented a substantial proportion of the overall fungal community, with Ascomycota and Basidiomycota accounting for 70.83 and 22.65%, respectively. The occurrence of Fusarium increased from 14.74% in TN1 to 18.45% in TN3. This rise in Fusarium prevalence may directly contribute to the incidence of mango root rot. The asexual reproduction of Fusarium is classified under the subphylum Hemiomicta, whereas its sexual reproduction phase falls within the Ascomycetes, posing a severe threat to numerous crops globally (Gálvez and Palmero, 2022; Zhang et al., 2022).

This study illustrates that soil-borne pathogens affect the microbial community architecture within the root systems, resulting in a diminished population of advantageous, disease-resistant microorganisms and a reduction in root defenses. Consequently, this situation precipitates the occurrence of plant diseases. Additionally, an evident negative correlation was observed when analyzing the *α*-diversity indices of fungal and bacterial communities. The escalation of soil-borne disease severity is closely linked to the accumulation of rhizosphere-associated fungal microbes, indicating that the majority of soil borne diseases affecting mango crops are predominantly fungal in nature.

### 4.4 The relationship between microbial community and soil physicochemical properties

The roots of plants play a vital role in the absorption of nutrients and water, while the composition of the microbial community associated with these roots significantly affects nutrient transformations and the efficiency of nutrient uptake by plants. These microorganisms possess the ability to adapt by utilizing root secretions to alter their surroundings, thereby enhancing nutrient absorption processes. Consequently, gaining insights into the interactions between soil microorganisms, the physical and chemical characteristics of the soil, and root exudates is essential for elucidating the mechanisms underlying soil borne diseases.

The RDA of microbial communities at the phylum level indicates that both Bacteroidota and Actinobacteriota markedly enhance the concentrations of AP, AN, AK, TK, and OM in the soil. Bacteroidota, in particular, is recognized as a key contributor to the phosphorus and nitrogen levels in the soil (Yousuf et al., 2012). Actively engaging in the organic matter cycle through the mineralization of various organic compounds (Pan et al., 2023). Actinobacteriota play a vital role in nutrient cycling by utilizing potassium and phosphorus present in the soil. They significantly contribute to the decomposition of organic matter, as well as the cycling of key soil elements such as carbon, nitrogen, and phosphorus (Boubekri et al., 2022). The redundancy analysis (RDA) of fungal communities at the phylum level indicated that Basidiomycota and Chytridiomycota markedly increased the concentrations of TK, TP, OM, and AN in the soil. This enhancement is likely attributed to the decomposition of plant litter, which facilitates the accumulation of both organic matter and inorganic salts. Basidiomycota, recognized as one of the most diverse and abundant phyla of soil fungi, serves as the principal decomposer within soil ecosystems (He et al., 2022). The findings of this study align with those of Zhu et al. (2021), both confirming a positive correlation between soil Basidiomycota and the levels of soil carbon and nitrogen.

Numerous investigations have demonstrated that the buildup of phenolic acids in the soil significantly contributes to challenges associated with continuous cropping (Canarini et al., 2019; Meng et al., 2022). A strong relationship has been identified between phenolic acids and soil microorganisms present in the rhizosphere. Within the bacterial community, Chloroflexi and WPS-2 show a positive correlation with syringic acid, cinnamic acid, and benzoic acid originating from root exudates, indicating that these phenolic compounds released by the roots modify the microbial community structure. Chloroflexi and WPS-2 not only preferentially utilize these root exudates but also possess the ability to degrade them, thereby alleviating soil borne diseases they may induce. Additional research into their biological functions is warranted.

In the fungal community, Basidiomycota displays a positive correlation with ferulic acid, Ascomycota associates with benzoic acid, and Chytridiomycota is linked to the majority of the phenolic acids identified in root exudates. Chytridobacteria exhibited the strongest correlation with gallic acid found in root secretions. It is speculated that phenolic acids produced in the mango rhizosphere exert a more significant influence on the fungal community compared to the bacterial community, thus facilitating the growth of pathogenic fungi. This growth is a critical contributing factor to the incidence of soil borne diseases affecting mangoes.

### 4.5 Changes in microbial community function

The KEGG database encompasses a diverse range of functional genes essential for comprehending cellular and organismal functionality from both a comprehensive and genomic viewpoint. Our analysis revealed that the functional gene profiles were consistent across samples, encompassing genes essential for fundamental metabolic processes, including energy metabolism, amino acid metabolism, and carbohydrate metabolism, collectively referred to as “housekeeping genes.” In contrast, the severe disease cohort (TN3) exhibited significant disparities in 14 KEGG secondary pathways, such as membrane transport, amino acid metabolism, and transport and catabolism, when compared to TN1 and TN2. The occurrence of soil borne diseases causes changes in microbial community structure, leading to differences in gene function, which is consistent with the experimental results of Yan et al. (2024). The enriched physico-chemical properties of the soil in TN3 are indicative of a heightened metabolic capacity among microorganisms. Nevertheless, the specific forms of these genes within the soil matrix and their operational mechanisms remain to be elucidated.

## 5 Conclusion

The composition of the microbial community, the physicochemical properties of the soil, and the root exudates of mango trees were examined following varying degrees of impact from soil borne diseases. Furthermore, we analyzed the correlation between alterations in the microbial community and environmental factors, as well as predicted shifts in bacterial community functionality. Our findings indicate that soil borne diseases diminish the roots’ efficiency in nutrient uptake, resulting in nutrient accumulation within the soil. Mango soil borne diseases secrete vanillic acid, salicylic acid, benzoic acid, and other phenolic compounds, with salicylic acid displaying the highest concentration. These phenolic compounds have induced significant alterations in the microbial community structure. The *α*-diversity index of bacteria has decreased, while the α-diversity index of fungi has increased, indicating a transition from bacterial to fungal dominance. Notably, there has been a decrease in the population of Acidobacteriota alongside a marked increase in Fusarium species. The functionality of the microbial community experiences considerable shifts post-disease onset, leading to an imbalanced microbial structure that compromises disease resistance. Overall, this investigation addressed the mechanisms underlying soil borne diseases through the lenses of soil physicochemical properties, root exudates, and microbial interactions, potentially aiding in the mitigation of challenges associated with mango’s soil borne diseases.

## Data Availability

The resultant raw reads were submitted to the NCBI Sequence Read Archive (SRA) database under Accession Number: PRJNA 1170051.

## Footnotes

^1^ http://drive5.com/uparse/

^2^ http://rdp.cme.msu.edu/

^3^ http://www.cytoscape.org

^4^ http://bioinformatics.psb.ugent.be/webtools/Venn

^5^ http://picrust.github.io/picrust/tutorials/genome_prediction.html
