# Supplementary material for: Root exudates and microbial community structure characteristics of mango under soil borne diseases
Source: Front Microbiol. 2025 Jul 10;16:1627112. doi: 10.3389/fmicb.2025.1627112 (PMC12287067; doi:10.3389/fmicb.2025.1627112)

Supplementary Material


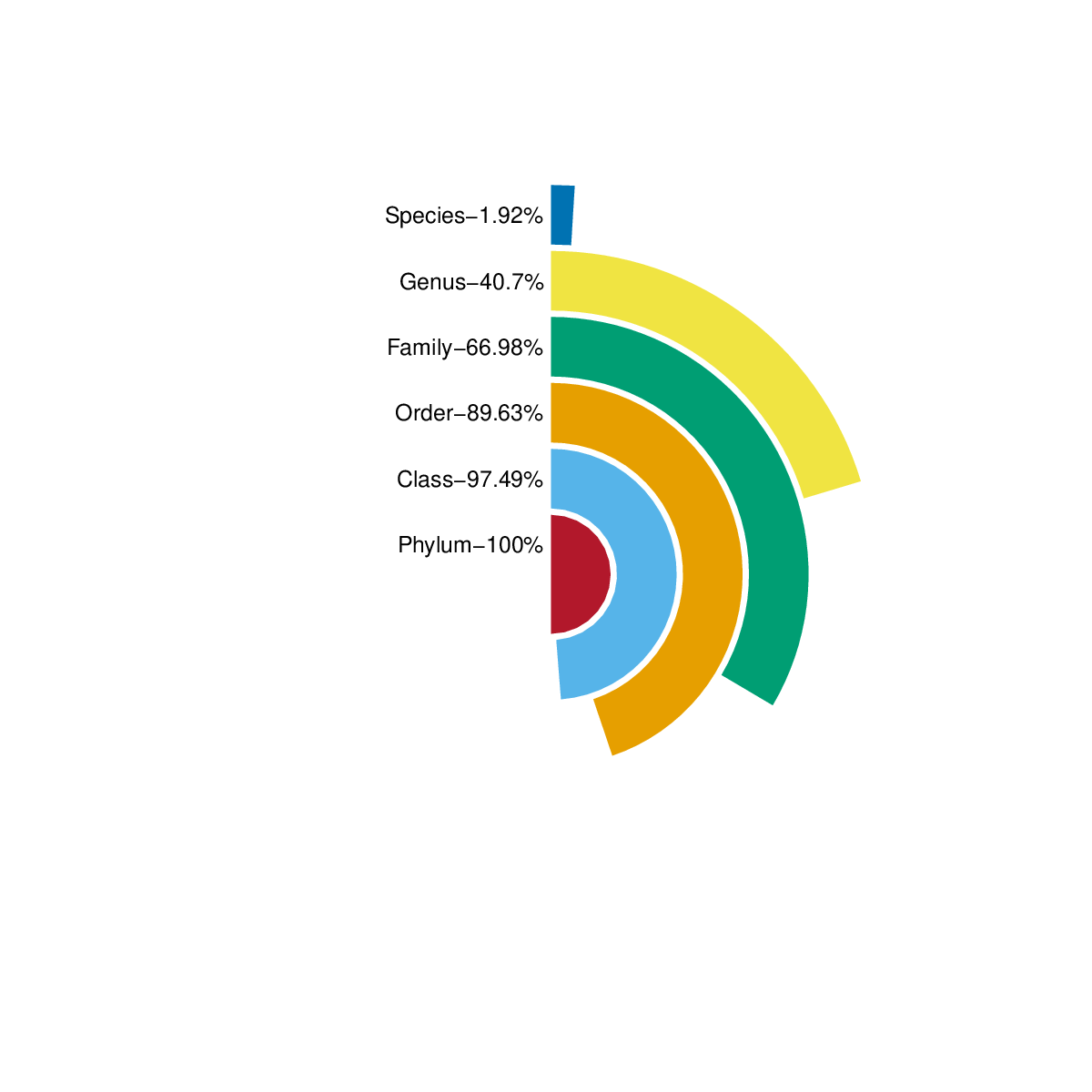
**Figure 1.** Bacterial annotation ratio chart

**Note**: Use a circular ring to display the proportion of all samples that have been successfully annotated at different taxonomic levels. The circular rings, from the inside out, represent phylum, class, order, family, and genus in a horizontal sequence. The semi-circle represents 100% annotation.


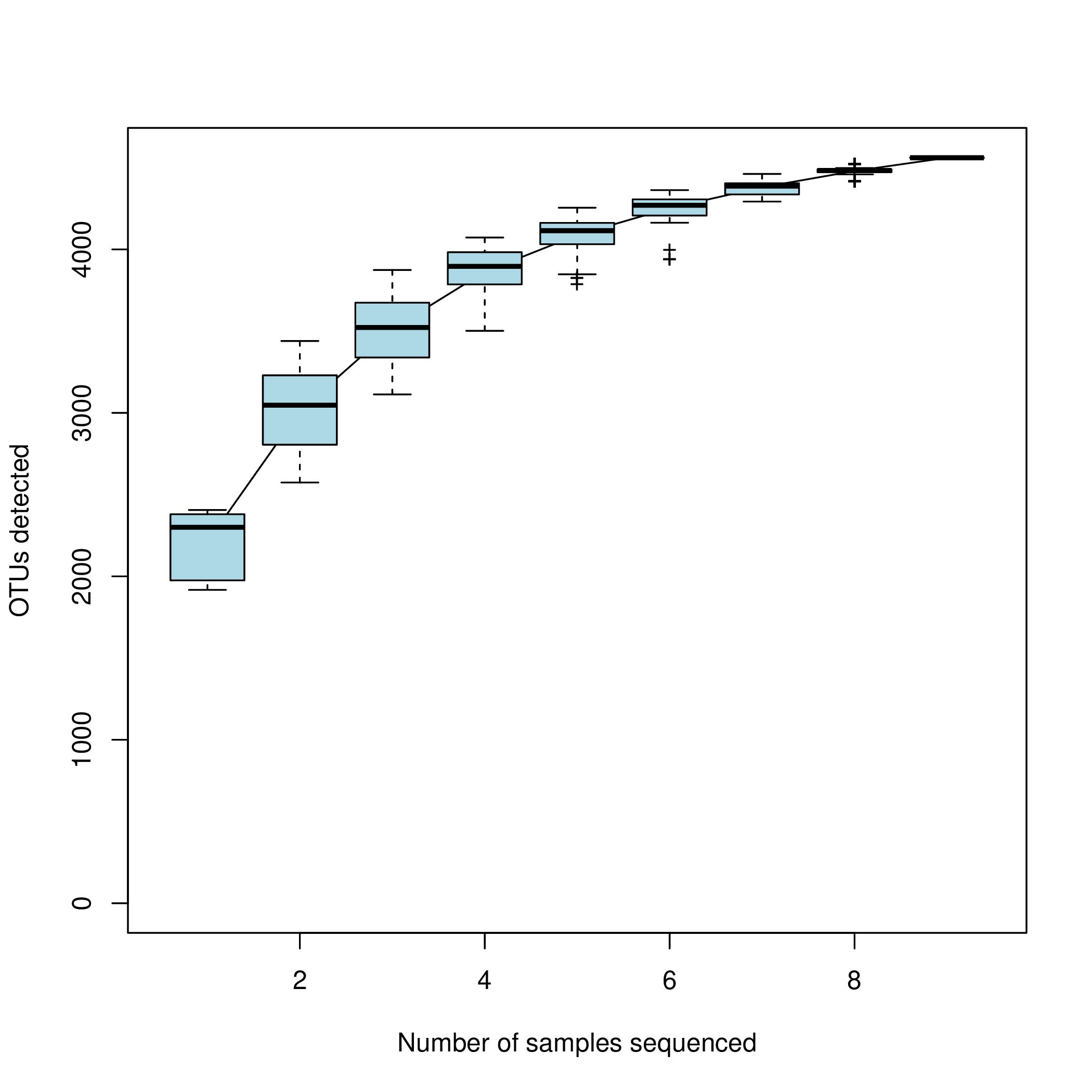

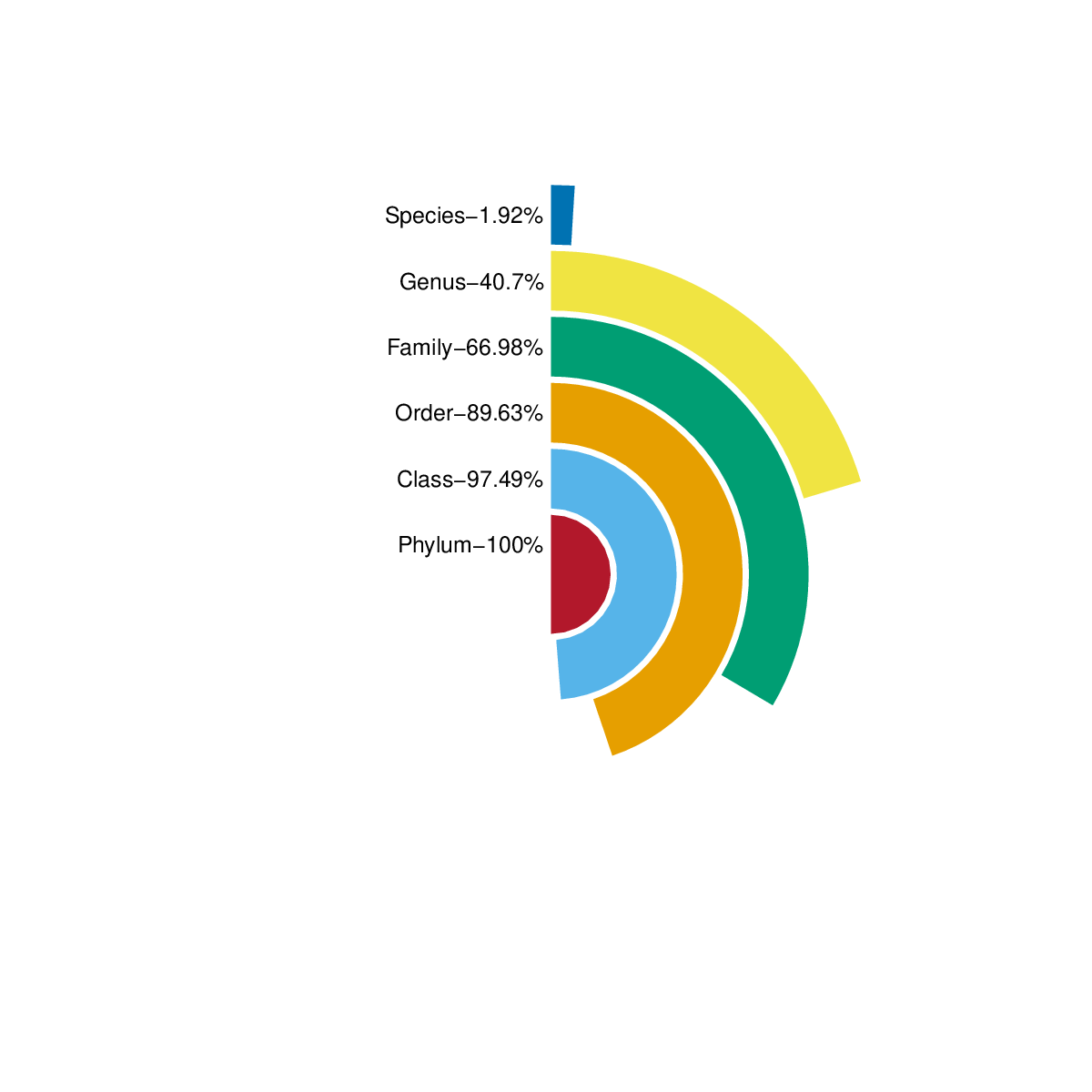
**Figure 2.** Bacterial species accumulation curve graph

**Note:** The horizontal coordinate represents the sample size; The vertical coordinate represents the number of OTUs after sampling. The results reflect the rate of emergence of new OTUs (new species) under continuous sampling. Within a certain range, as the sample size increases, if the curve shows a sharp rise, it indicates that a large number of species have been discovered in the community. When the curve tends to level off, it indicates that the species in this environment will not increase significantly with the increase of the sample size. The species accumulation curve can be used as a judgment on whether the sample size is sufficient. A sharp increase in the curve indicates that the sample size is insufficient and the sampling size needs to be increased. Conversely, it indicates that the sampling is sufficient and data analysis can be conducted.


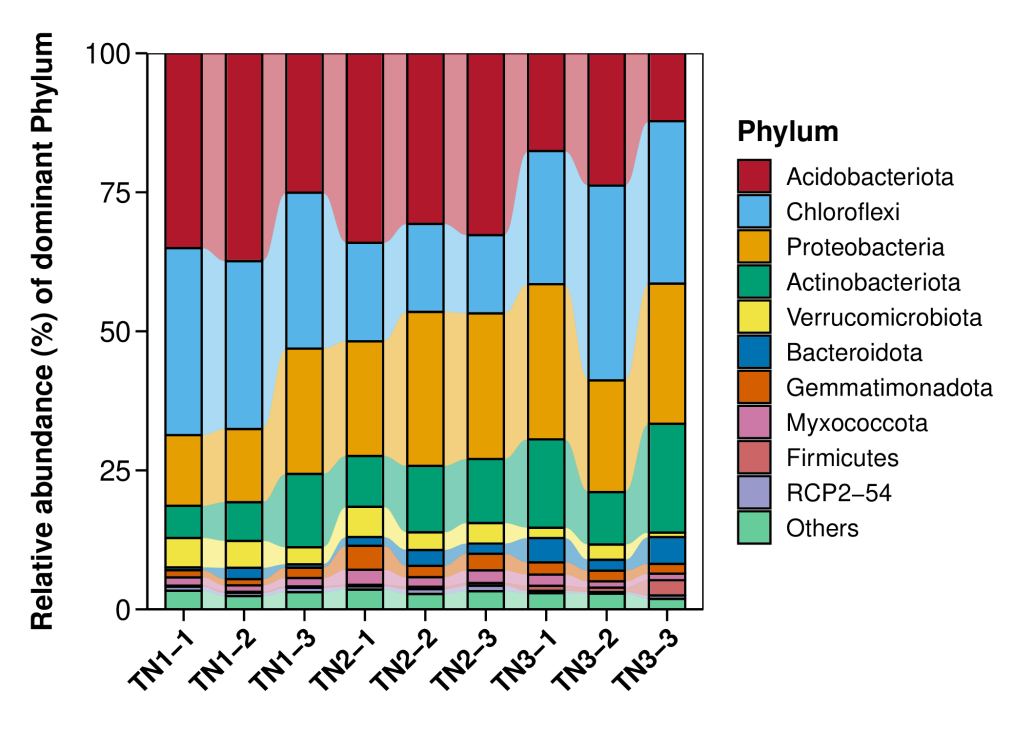


**Figure 3.** Bacterial phylum sample accumulation impact diagram

**
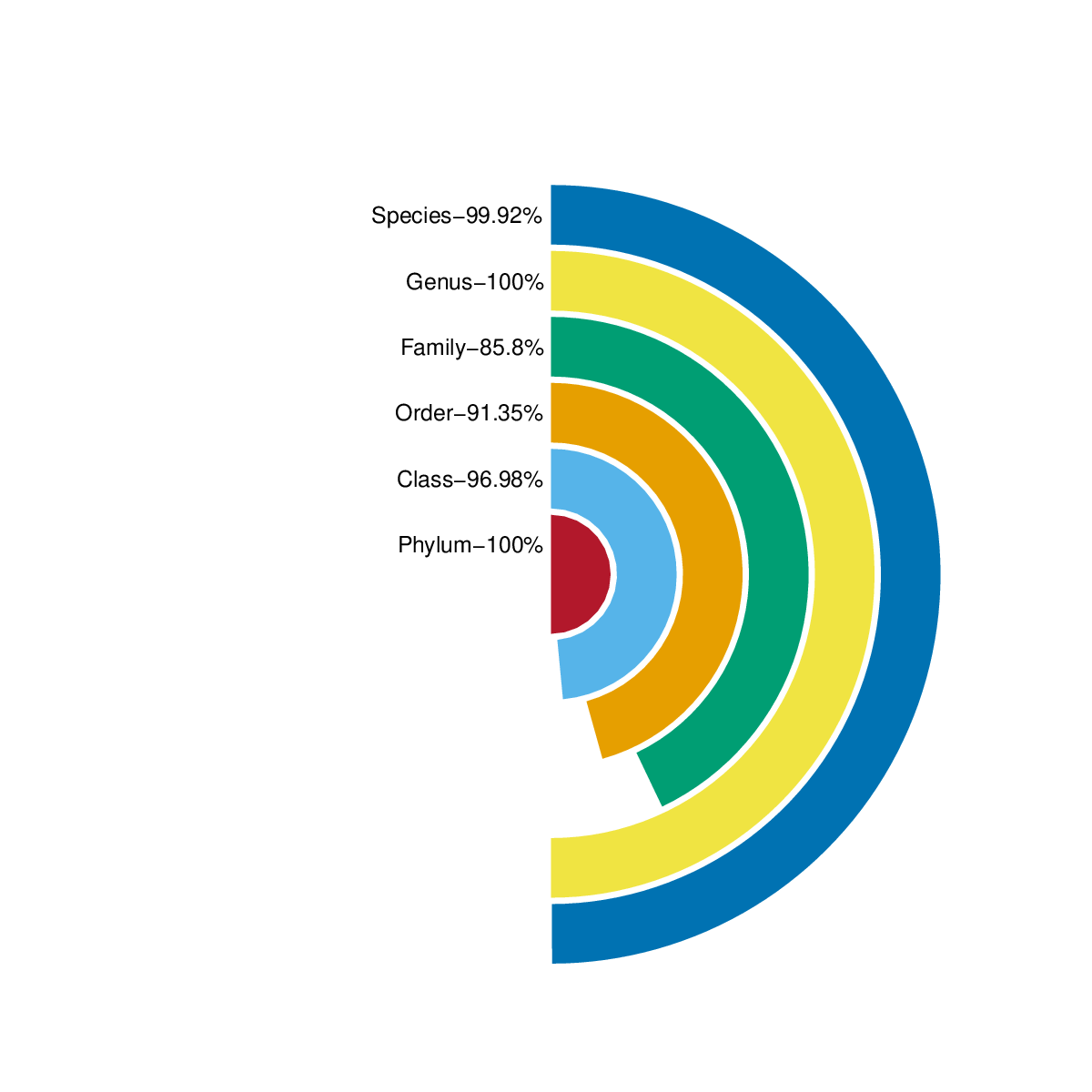
**


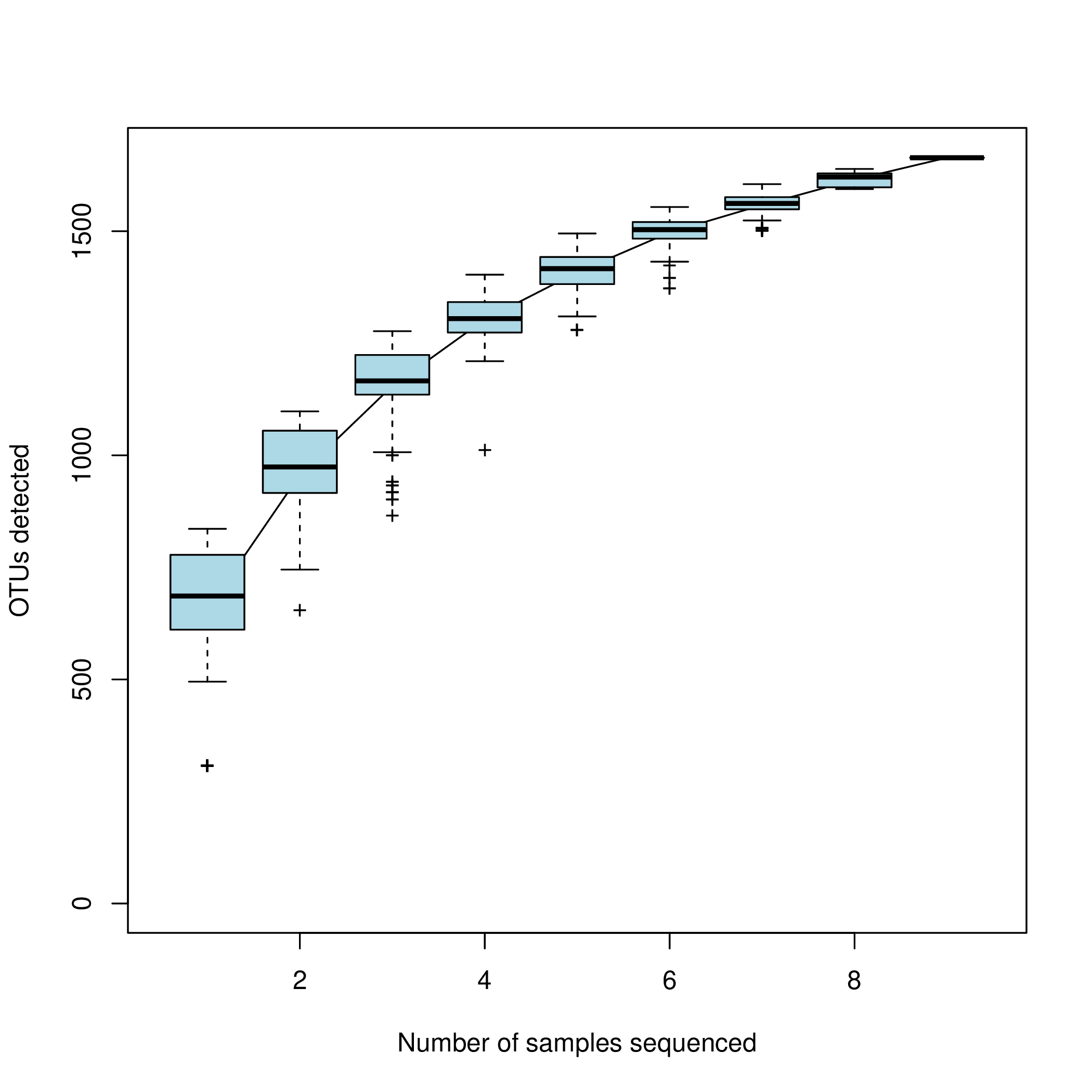
**Figure 4.** Fungal annotation ratio chart

**Figure 5.** Fungal species accumulation curve graph

**Figure 6.** Fungal phylum sample accumulation impact diagram
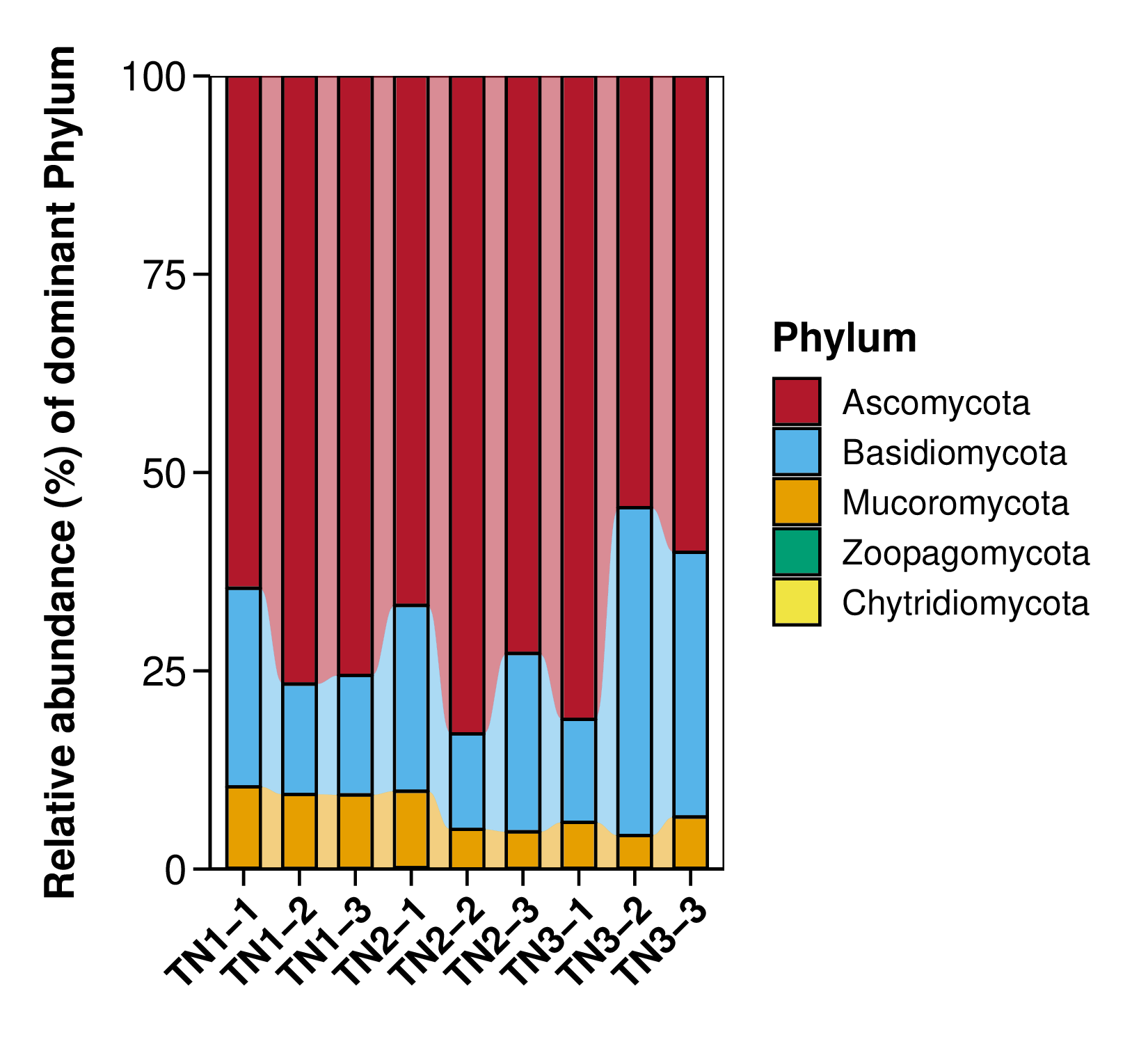

Supplement: Supplementary file 1 [file Table_1.DOCX]
